# Spiky mesoporous silica-based nanovaccines enhance mucosal immunity with improved intestinal retention and antigen accessibility

**DOI:** 10.1016/j.mtbio.2026.103391

**Published:** 2026-07-01

**Authors:** Lin Yu, Xiaofeng Shi, Yaming Yu, Gaofeng Cai, Xuping Zhao, Fangzhu Lin, Xuanqi Lu, Jie Qiao, Yushan Fang, Kexin Fang, Yang Yang, Deyun Wang

**Affiliations:** a Institute of Traditional Chinese Veterinary Medicine, College of Veterinary Medicine, Nanjing Agricultural University, Nanjing, 210095, PR China; b Jiangxi Provincial Key Laboratory for Animal Health, College of Animal Science and Technology, Jiangxi Agricultural University, Nanchang, Jiangxi, 330045, PR China

**Keywords:** Virus-inspired MSNs, Oral nanovaccine, *Rehmannia glutinosa* polysaccharide, Intestinal infections

## Abstract

Intestinal vaccination faces several key challenges, including limited epithelial interaction, inefficient antigen uptake, and insufficient immune stimulation. Here, we developed a virus-inspired oral nanovaccine based on spiky mesoporous silica nanoparticles (VMSNs) functionalized with *Rehmannia glutinosa* polysaccharide (RGP), a natural polysaccharide with favorable intestinal biocompatibility. The resulting RGP-VMSN showed enhanced intestinal retention and epithelial interaction and was associated with increased antigen accessibility within the mucosal environment, reflected by stronger antigen-associated signals at mucosal immune sites. Using ovalbumin (OVA) as a model antigen, oral OVA@RGP-VMSN vaccination was associated with increased intestinal dendritic cell activation, enhanced germinal center responses, and elevated antigen-specific antibody production, together with coordinated mucosal and systemic immune responses. Furthermore, immunization with a *Salmonella enterica* serovar Typhimurium lysate-loaded nanovaccine improved protection in a murine challenge model. Overall, this strategy represents a promising platform for oral and mucosal nanovaccine development.

## Graphical abstract

**Fig. 1**. Contribution of the OVA@RGP-VMSN nanovaccine to enhance mucosal and systemic immune responses.(A) Schematic illustration of the synthesis of OVA@RGP-VMSN nanoparticles. (B) Following oral administration, OVA@RGP-VMSN is internalized by antigen-presenting cells (APCs). (C) Oral OVA@RGP-VMSN vaccination is accompanied with immune responses in Peyer's patches and mesenteric lymph nodes (MLNs), including dendritic cell activation and germinal center responses. (D) Systemic protection is associated with activation of memory T and B cells in the spleen and secretion of antigen-specific antibodies into the serum. Figure created using BioRender.

**Figure:**
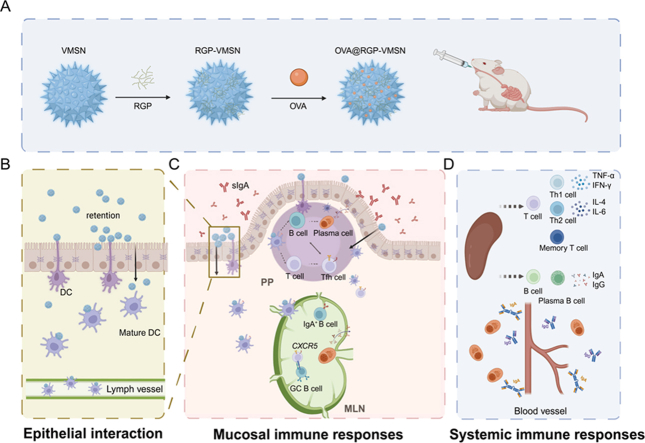

## 1 Introduction

Intestinal mucosal immunity is the primary defense against intestinal pathogens [1]. Approximately 70% of pathogenic microorganisms enter the body via mucosal surfaces, which serve as both protective barriers and major entry sites for pathogens [2]. The gastrointestinal tract maintains a balance among nutrient absorption, barrier integrity, immune tolerance, and immune activation [[3], [4], [5]]. Importantly, this mucosal environment contains the architecture required to initiate adaptive immune responses. Once antigens reach these inductive sites, antigen-specific secretory immunoglobulin A (SIgA) responses can be generated at mucosal surfaces along with systemic IgG responses. In addition, mucosal immune responses can disseminate to distant mucosal tissues through the common mucosal immune system (CMIS) [6,7]. SIgA neutralizes pathogens before invasion, prevents colonization, and blocks systemic spread [8], whereas IgG protects deep tissues and the circulatory system [9]. This dual protection, together with needle-free delivery, high patient acceptance, and suitability for mass vaccination in resource-limited settings, makes oral immunization an attractive strategy [10]. However, gastric acid, bile salts, digestive enzymes, mucus, and epithelial tight junctions degrade or block most antigens before they reach inductive sites, resulting in weak and inconsistent oral vaccine efficacy [11].

To reduce antigen degradation, nanomaterial-based carriers such as mesoporous silica and other inorganic or organic nanoplatforms have been increasingly been used to stabilize antigens and improve gastrointestinal transit [[12], [13], [14]]. However, improved stability alone is insufficient. Most nanoformulations still face the challenge of accessing or traversing the epithelium. Self-assembled nanoparticles typically adopt low-energy spherical shapes with limited surface anchoring sites and limited interfacial roughness, resulting in weak mucosal adhesion and poor epithelial uptake [15,16]. This limitation represents a major bottleneck that restricts the transition from merely “protecting the antigen” to effectively “introducing the antigen into immune circuits” and may partly explain why many oral nanovaccines underperform despite favorable physicochemical properties. Recent studies on dendritic cell (DC)-targeted biomaterials and nanovaccines further suggest that effective immune induction depends not only on antigen protection but also on efficient antigen exposure to antigen-presenting cells (APCs), internalization, and immune responses in lymphoid tissues [17]. Beyond functioning solely as carrier stabilizers, material properties such as surface structure, shape, and mechanical features may influence the spatial exposure of antigen-associated signals near APCs and mucosal immune sites, thereby affecting antigen accessibility, cellular uptake, and immune priming [18].

Nature provides inspiration for architectural design, particularly through viral nanoscale surface features such as spikes, rough textures, and varied curvature that facilitate biological barrier crossing [19]. Multivalent protrusions on viral surfaces enhance membrane binding, frictional coupling, and energy-dependent endocytic interactions at biological interfaces [20,21]. Consequently, mimicking viral topology has emerged as a promising strategy to improve nanomaterial-epithelium interactions. Mesoporous silica nanoparticles (MSNs) are attractive candidates because of their structural rigidity, acid resistance, large surface area for antigen loading, and versatile surface chemistry [22]. Spiky or virus-mimicking MSNs (VMSNs) are particularly attractive because their nanoscale roughness and protrusions enhance epithelial interactions, resulting in stronger mucosal retention and more efficient cellular internalization than smooth spherical carriers [23,24]. These properties suggest that specific topologies may increase the likelihood that orally delivered antigens reach inductive mucosal sites. However, topology alone may be insufficient to support robust mucosal immune responses. Oral vaccines must also interact with the epithelial barrier and support immune responses in mucosal lymphoid tissues [25]. Therefore, immune signaling represents another critical dimension of oral vaccine design beyond epithelial access alone. An effective oral nanovaccine platform should ideally support epithelial interaction, antigen accessibility, APC activation, and adaptive immune responses in a coordinated manner [26].

Certain natural plant polysaccharides have been reported to enhance immune function [27,28]. These compounds are safe for oral administration, biocompatible, and recognized immunomodulators. *Rehmannia glutinosa* polysaccharide (RGP) has been shown to influence intestinal immunity and promote DC maturation [[29], [30], [31]]. Our previous work identified the polysaccharide composition of RGP and confirmed its biological activity following oral administration [32]. Molecular weight analysis indicated an Mw of 145.821 kDa and an Mn of 64.059 kDa, and monosaccharide composition analysis showed that RGP was primarily composed of fucose, rhamnose, arabinose, galactose, glucose, xylose, and mannose at a molar ratio of 1.05:18.98:28.12:30.54:12.60:3.26:5.49 [33]. Owing to its favorable tolerability and relatively low reactogenicity, RGP was selected as an orally compatible immunomodulatory adjuvant for incorporation into the immune delivery platform [34].

In this study, we developed a virus-mimicking oral nanovaccine platform based on spiky MSNs functionalized with RGP (RGP-VMSN). The combination of spiky nanoscale topology and RGP functionalization was associated with enhanced epithelial interaction, increased antigen-associated signals in mucosal lymphoid tissues, and coordinated activation of DC-related and adaptive immune responses.

Our findings show that RGP-VMSN is associated with increased DC activation in the intestinal mucosa and greater antigen-associated signals in draining mesenteric lymph nodes (MLNs). To evaluate the ability of this platform to induce mucosal immune responses, ovalbumin (OVA) was first used as a model antigen to characterize antigen distribution and immune activation. To further assess the potential applicability of the same carrier design in a pathogen-related setting, a pathogen-relevant antigen system was subsequently established using *Salmonella* Typhimurium lysate (STL), and protective efficacy was evaluated in a *Salmonella* challenge model. Under the experimental conditions used in this study, the STL-loaded platform improved protective outcomes in a *Salmonella* challenge model.

Overall, the integrated platform was associated with enhanced epithelial interaction and mucosal immune activation, supporting its potential as a design strategy for oral nanovaccines.

## 2 Materials and methods

### 2.1 Materials

Tetraethyl orthosilicate (TEOS, 98%), ethylenediamine (≥99.5%), formaldehyde solution (37 wt%), 3-aminophenol (3-AP, 98%), OVA (≥98%), ammonium hydroxide solution (28-30%) and rhodamine B isothiocyanate (RBITC) were purchased from Aladdin Reagent Co., Ltd. (Shanghai, China). 1-Ethyl-3-(3-dimethylaminopropyl) carbodiimide hydrochloride (EDC·HCl), N-hydroxysuccinimide (NHS), and 3-aminopropyltriethoxysilane (APTES) were obtained from Macklin Biochemical Technology Co., Ltd. (Shanghai, China). Simulated body fluid (SBF), simulated intestinal fluid (SIF), and simulated gastric fluid (SGF) were purchased from Shanghai Yuanye Bio-Technology Co., Ltd. (Shanghai, China). FITC-conjugated anti-OVA antibodies and flow cytometry (FCM) antibodies were obtained from Thermo Fisher Scientific (Waltham, MA, USA) and HRP-labeled secondary antibodies were from ABclonal (Wuhan, China). Detailed antibody information is provided in Table S1 of the Supplementary Materials. ELISA kits were obtained from Multi Sciences (Nanjing, China), and the BCA protein assay kit was purchased from Beyotime Biotechnology (Shanghai, China). All other chemicals were of analytical grade.

### 2.2 Extraction of RGP and preparation of OVA@RGP-VMSN

RGP was obtained from Shanxi Ciyuan Biotechnology Co., Ltd. (Shanxi, China, batch number: CY20220306) through ethanol defatting, hot-water extraction, ethanol precipitation, and deproteinization. Detailed structural characterization is provided in the Supporting Information.

VMSN was synthesized using a modified Stöber method [35]. Briefly, 3-AP and formaldehyde were reacted in an anhydrous ethanol/ammonium hydroxide/ethylenediamine system to form an aminophenol-formaldehyde (APF) core. TEOS and 3-AP were subsequently added to generate spiky VMSN particles. These particles were centrifuged, washed, and dried, followed by amination with APTES and covalent conjugation with EDC/NHS-activated RGP to obtain RGP-VMSN. OVA was then physically adsorbed onto RGP-VMSN at 4°C, and antigen loading content and adsorption efficiency were measured using the BCA assay. Antigen loading efficiency was calculated as follows:

$$\text{LE}\;\left(\%\right)=\frac{{OVA}_{total}-{OVA}_{free}}{{OVA}_{total}}\times 100\%$$

RGP and OVA used for vaccine preparation were passed through Pierce Endotoxin Removal Columns (Thermo Scientific, Illinois, USA) before formulation. Endotoxin levels in the final OVA-based vaccine formulations were measured using a Limulus amebocyte lysate (LAL) assay, and the endotoxin level of each administered vaccine dose was confirmed to be below 0.1 EU.

### 2.3 Characterization of nanoparticles

VMSN morphology was examined using transmission electron microscopy (TEM; Tecnai G2 F30, FEI, USA). Hydrodynamic diameter and zeta potential were measured using dynamic light scattering (Malvern Zetasizer Nano ZS90). Nitrogen adsorption–desorption isotherms were recorded using a BET surface area analyzer (Micromeritics ASAP 2460; Micromeritics, Norcross, GA, USA) and specific surface area and pore size distribution were calculated using the BET and BJH models, respectively. Fourier-transform infrared (FTIR) spectra were collected using a Nicolet iS1 spectrometer (Thermo Scientific) to confirm polysaccharide functional groups and covalent coupling. Thermogravimetric analysis (TGA) was performed using a STA449 instrument (Netzsch, Selb, Germany) to quantify organic content and grafting amount. X-ray photoelectron spectroscopy (XPS) was conducted using a K-Alpha system (Thermo Fisher Scientific) to analyze elemental composition and chemical bonding states. X-ray diffraction (XRD) patterns were recorded on a SmartLab diffractometer (Rigaku, Tokyo, Japan) over 5°–45° (2θ) to assess the structural features of the silica framework. Static water contact angles were measured with a JC2000D1 goniometer (Zhongchen Digital Technology, Shanghai, China) to evaluate surface hydrophilicity.

### 2.4 In vitro release and stability

OVA@RGP-VMSN and OVA@VMSN were incubated in SGF, (pH 1.2, without pepsin) and SIF (pH 6.8, without trypsin) at 37°C. At designated intervals, samples were centrifuged (8000 rpm, 4°C), and protein concentrations in the supernatants were quantified using the BCA assay. Equal volumes of fresh medium were added after each sampling. Antigen release kinetics were calculated from the cumulative protein release.

For stability analysis, particles were incubated in SGF (pH 1.2, with pepsin) and SIF (pH 6.8, with trypsin). Structural integrity was evaluate using TEM, whereas size distribution and polydispersity index (PDI) were measured using DLS. The secondary structure of OVA was assessed using circular dichroism spectroscopy (Bio-Logic MOS 450, France) to evaluate conformational stability. Particle degradation was also examined using TEM after incubation in SBF.

### 2.5 Cell lines and animals

Caco-2 cells obtained sourced from Yizefeng Biotechnology Co., Ltd (Shanghai, China), were cultured in RPMI-1640 medium supplemented with 10% fetal bovine serum and 1% penicillin-streptomycin. Bone marrow-derived dendritic cells (BMDCs) were prepared as previously described and cultured under the same conditions [22].

Female BALB/c mice (4-5 weeks old) were obtained from the Yangzhou University Laboratory Animal Center. Animals were maintained under specific pathogen-free (SPF) conditions at 21 ± 1°C with 40%-70% humidity and a 12 h light/dark cycle and had free access to food and water. All experimental procedures were approved by the Animal Ethics Committee of Nanjing Agricultural University (approval number: 2016YFD0500502).

### 2.6 Preparation of RBITC-labeled VMSN and RGP-VMSN

RBITC-labeled nanoparticles were prepared according to a previously reported method [12]. Briefly, 100 mg of RBITC was dissolved in 2 mL of anhydrous ethanol, followed by the addition of 100 μL of APTES. After stirring for 24 h in the dark, 400 μL of the obtained RBITC-APTES solution was added to 80 mg of VMSN or RGP-VMSN dispersed in 8 mL of anhydrous ethanol. The mixture was reacted for another 24 h in the dark. The nanoparticles were then collected by centrifugation, washed repeatedly with anhydrous ethanol until no visible fluorescence remained in the supernatant, dried, and stored protected from light.

### 2.7 Evaluation of cellular uptake and exocytosis-related pathways

Caco-2 cells were cultured in 6-well plates and pretreated for 1 h at 37°C with chlorpromazine (CPZ; 30 μM), methyl-β-cyclodextrin (MβCD; 2 mM), 5-(N-Ethyl-N-isopropyl) amiloride (EIPA; 10 μM), or chloroquine (30 μg/mL). RBITC-labeled nanoparticles (125 μg/mL) were then added and incubated for 2 h. After washing, cells were harvested and analyzed using FCM to determine the percentage of RBITC-positive cells.

For exocytosis studies, cells were pre-incubated with the labeled nanoparticles for 2 h and then washed to remove free particles. Cells were then incubated in serum-free medium containing brefeldin A (25 μg/mL), bafilomycin A1 (100 nM), or monensin sodium (32.5 μg/mL) for 3 h. Intracellular fluorescence was analyzed using FCM to evaluate the exocytosis pathways.

### 2.8 In vivo biodistribution in GIT and MLNs

Cy5.5-labeled OVA was loaded into VMSN or RGP-VMSN and orally administered orally at 20 mg/kg. Gastrointestinal tract samples, MLNs and major organs were collected at 1, 2, 4, 6, 8, 12, and 24 h after administration. Fluorescence images were acquired using an IVIS Spectrum imaging system (PerkinElmer, Waltham, MA,USA) and quantified with Living Image® 4.3.1 software.

To evaluate the presence and distribution of antigen-associated signals in MLNs, FITC-OVA and RBITC-labeled RGP-VMSN were orally administered. MLNs were collected after 4 h post-administration for cryosectioning, DAPI staining, and fluorescence microscopy to assess the spatial association of FITC and RBITC signals in MLN sections.

For epithelial interaction analysis, ileal tissues were collected and stained for CD11c (Cy5), OVA (FITC), and nuclei (DAPI). Spatial association between nanoparticles and CD11c^+^ cells in the intestinal mucosa was examined. Nanoparticle interactions with villi and epithelial junctions were further analyzed using TEM.

### 2.9 Transwell co-culture and DC activation

Caco-2 cells were seeded onto 6-well Transwell inserts (Corning, NY, USA; polyester membrane, 0.4 μm pore size) at a density of 1.0 × 10^5^ cells per well and were cultured for 14 days to form a tight epithelial barrier. Medium in both apical and basolateral compartments was replaced every 48 h. Barrier integrity was confirmed using transepithelial electrical resistance (TEER) measurements (Millicell-ERS, Merck, USA), and monolayers with TEER ≥300 Ω/cm^2^ were used for experiments.

Equal doses of OVA (50 μg per insert) in different formulations (OVA, RGP + OVA, OVA@VMSN and OVA@RGP-VMSN) were added to the apical chamber. After 4 h, membranes were stained with Cy5-conjugated anti-CD11c and FITC-conjugated anti-Occludin antibodies and were imaged using 3D confocal microscopy (Nikon A1R confocal microscope; 63× oil immersion objective, z-step 0.5 μm).

For BMDC assays, BMDCs were seeded in the basolateral chamber. Following co-culture with different formulations, basolateral BMDCs were collected at the indicated time points and analyzed using FCM to determine the frequency of FITC-OVA^+^ CD11c^+^ cells. At 24 h, BMDCs were stained for CD80, CD86, CD40, and MHC-II, and cytokine levels (IL-6, TNF-α, and IL-1β) in basolateral supernatants were measured by ELISA.

### 2.10 Immunization schedule

Female BALB/c mice were randomly divided into 5 groups (n = 20 per group): control, OVA, RGP + OVA, OVA@VMSN, and OVA@RGP-VMSN. The control group received 0.5 mL of water by oral gavage, whereas the remaining groups received 0.5 mL of the corresponding formulation containing 100 μg OVA. Mice were orally immunized on days 1–3, 14, and 21.

MLNs were collected at 24 and 48 h after the first immunization to analyze CD11c^+^ cell and T-cell activation, and on day 10 to characterize germinal center (GC) B cells and T follicular helper (Tfh) cells. MLNs were harvested on day 28 to evaluate B cell differentiation, including IgA^+^ B cells and plasma cells. Spleens were collected on day 28 for T cell phenotypic and functional analyses. Serum samples were collected on days 14, 21, 28, 42, and 60 to measure OVA-specific antibodies and subclasses. Intestinal lavage fluid, bronchoalveolar lavage fluid, and vaginal wash samples were collected on day 28 to quantify OVA-specific IgA. Major organs were harvested on day 60 for histological evaluation.

### 2.11 Mucosal immune analysis

MLN cells were analyzed using FCM for CD11c^+^ cell (CD40, CD80, CD86, and MHC-II) and T-cell activation (CD3, CD4, CD8, and CD69). GC B cells (B220, FAS, and GL7), Tfh cells (CD3, CD4, CXCR5, and PD-1), and plasma cells (B220 and CD138) were also characterized. Detailed antibody information is provided in Table S1.

### 2.12 Systemic immune analysis

Serum OVA-specific IgG, IgG1, and IgG2a were quantified using ELISA following a previously reported method [36]. Briefly, plates were coated with OVA overnight, blocked with bovine serum albumin, incubated with serum samples, and detected using HRP-conjugated goat anti-mouse secondary antibodies. Tetramethylbenzidine (TMB) substrate was added for color development, and absorbance was measured.

Splenocytes were isolated to evaluate T-cell responses. After red blood cell lysis, cells were stained with antibodies against CD3, CD4, CD8, CD44, CD62L, CD69, CD107a, CD178, FOXP3, IFN-γ, TNF-α, IL-4, IL-10, and IL-17A and were analyzed using FCM. Cytokine levels were quantified using ELISA. Detailed antibody information is provided in Table S1.

### 2.13 Safety evaluation

Major organs were fixed in 4% paraformaldehyde and subjected to hematoxylin and eosin staining. Serum was simultaneously collected for biochemical analysis.

### 2.14 Challenge study and protective efficacy

*Salmonella enterica* serovar Typhimurium SL1344 *(S.* Typhimurium*)* was obtained from Biobw Biotechnology Co., Ltd. (Beijing, China; catalog no. BIO-110527). STL was prepared as follows. Bacterial suspensions with OD_580_ values of 0.3–0.8 were collected, washed, ultrasonicated and centrifuged. Supernatants were ultracentrifuged, endotoxins were reduced using Pierce Endotoxin Removal Columns (Thermo Scientific, Illinois, USA) and endotoxin levels in the vaccine formulation were measured using LAL assay (GenScript, Piscataway, NJ, USA), and the endotoxin level of each administered vaccine dose was confirmed to be below 0.1 EU. Total protein concentration was determined by BCA assay.

Female BALB/c mice were immunized on days 1–3, 14, and 21. Serum and mucosal samples were collected on day 28 for ELISA analysis of STL-specific IgG, IgG subclasses, and SIgA. Ten days after the final vaccination, mice were orally with 1 × 10^9^ CFU of *S.* Typhimurium. Survival was monitored twice daily and analyzed using the log-rank (Mantel–Cox) test in GraphPad Prism 9.0.

### 2.15 Statistical analysis

Data are presented as mean ± SD. Statistical analyses were performed using GraphPad Prism 9.0. One-way ANOVA followed by Tukey's multiple comparisons test was used for comparisons among multiple groups at a single time point. Two-way ANOVA followed by Tukey's multiple comparisons test was used for experiments involving two independent variables, such as treatment group and time. Exact *P*-values are reported in the figure legends. A *P* < 0.05 was considered statistically significant.

## 3 Results and discussion

### 3.1 Synthesis and surface functionalization of VMSN with RGP

**Fig. 1:**
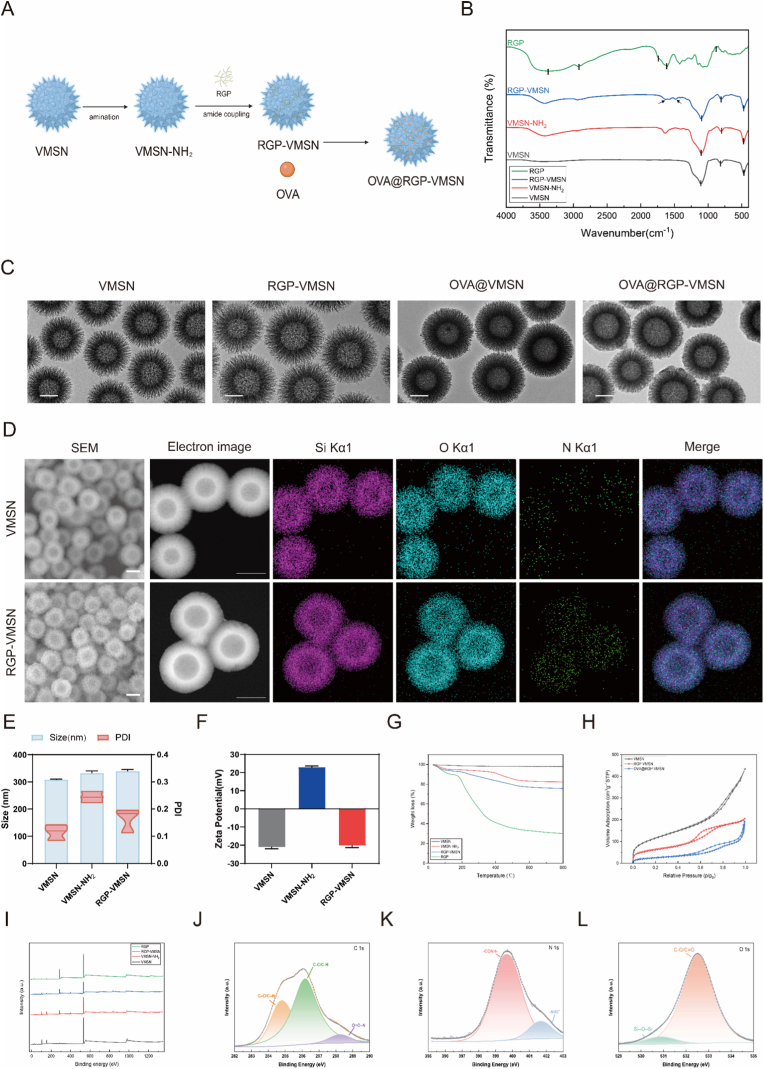
(A) Schematic illustration of the fabrication process of VMSN, RGP-VMSN, and OVA@RGP-VMSN. (B) FTIR spectra of VMSN, VMSN-NH_2_, RGP-VMSN and RGP. (C) TEM images of VMSN, RGP-VMSN, OVA@VMSN, and OVA@RGP-VMSN. Scale bars, 100 nm (D) SEM images, electron micrographs, and corresponding EDS elemental mappings (Si, O, N) of VMSN and RGP-VMSN. Scale bars in SEM and electron micrographs: 200 nm. (E) Particle size and PDI of nanoparticles. (F) Zeta potential of nanoparticles. (G) TGA curves and (H) nitrogen adsorption-desorption isotherms of nanoparticles. (I) XPS survey spectra of VMSN, VMSN-NH_2_, RGP-VMSN, and RGP. (J-L) High-resolution XPS spectra of C 1s, N 1s, and O 1s of RGP-VMSN. Data are presented as mean ± SD. (n = 3 independent experiments).

RGP-VMSN was synthesized using templated-assisted preparation and surface conjugation (Fig. 1A). TEM images showed that pristine VMSN exhibited a spherical morphology with dense spike-like protrusions, consistent with a virus-mimicking surface topology. After RGP modification, RGP-VMSN retained the overall spiky architecture, indicating that polysaccharide conjugation did not disrupt the characteristic surface morphology. Following OVA loading, both OVA@VMSN and OVA@RGP-VMSN displayed a peripheral low-electron-density layer around the particles, consistent with antigen adsorption on the mesoporous surface (Fig. 1C). DLS analysis showed a hydrodynamic of approximately 340 nm with a PDI below 0.3 (Fig. 1E).

FTIR analysis confirmed stepwise surface modfification. After amination, VMSN-NH_2_ showed characteristic absorption bands at 1620 cm^−1^ and 3400-3500 cm^−1^ [37]. The C=O stretching band at 1735 cm^−1^ was observed in both RGP and retained in RGP-VMSN [38]. Distinct amide I and amide II bands at 1650 cm^−1^ and 1550 cm^−1^ confirmed amide bond formation between RGP and the aminated surface [37]. The Si-O-Si and Si-O bands at 1070 cm^−1^ and 455 cm^−1^ remained unchanged [39], indicating preservation of the silica framework (Fig. 1B).

Elemental mapping revealed enhanced nitrogen signals after RGP modification (Fig. 1D). XPS survey spectra further confirmed successful surface modification, with RGP-VMSN showing stronger N signals than those of pristine VMSN (Fig. 1I). High-resolution XPS spectra identified characteristic N 1s (400.0 eV) and C 1s (288.2 eV) peaks associated with amide bonds (–NH–C=O/O=C–N), further supporting successful conjugation (Fig. 1J–L). Consistently, zeta potential shifted from −21.03 mV for VMSN to +22.9 mV after amination (VMSN-NH_2_), then decreased to −20.1 mV after RGP grafting (Fig. 1F).

XRD patterns of both materials showed broad amorphous silica peaks between 15° and 25°. A slight increase in signal intensity after RGP modification suggested local ordering without changes in crystallinity (Fig. S1A). TGA showed grafting amounts of 15.8% for amino and 22.9% for RGP (Fig. 1G). Nitrogen adsorption-desorption analysis showed type IV isotherms, and detailed parameters are summarized in Table S2. Sequential modification with RGP and OVA reduced the average pore size from 10.6 to 4.1 nm and the specific surface area from 421.13 to 91.11 m^2^ g^−1^, indicating effective surface coverage and pore occupation (Fig. S1B-F, Fig. 1H).

These findings confirm the successful construction of a structurally stable, virus-mimicking, and biofunctionalized RGP-VMSN platform suitable for subsequent antigen-loading and delivery studies.

### 3.2 Stability and release behavior in simulated gastrointestinal fluids

**Fig. 2:**
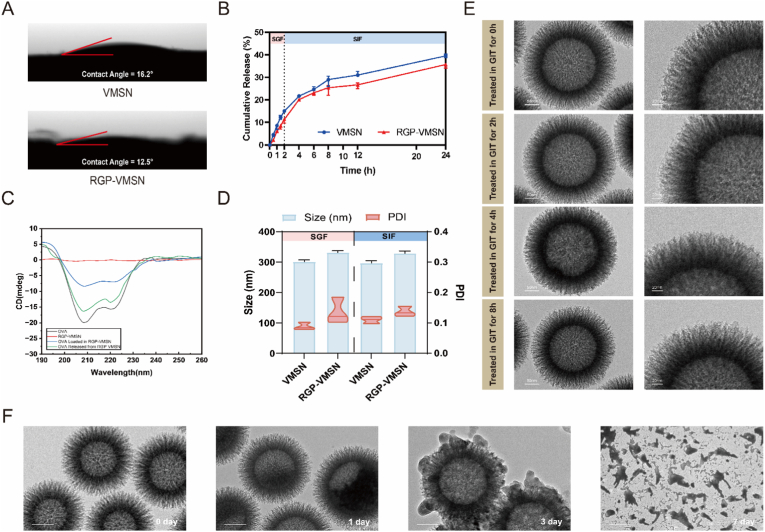
(A) Static water contact angles of VMSN and RGP-VMSN. (B) Cumulative OVA release profiles from VMSN and RGP-VMSN in SGF (without pepsin) and SIF (without trypsin) at 37°C. (C) CD spectra of OVA adsorbed onto and released from RGP-VMSN. (D) Particle size and PDI of VMSN and RGP-VMSN after incubation in SGF (with pepsin) and SIF (with trypsin). (E) TEM images of RGP-VMSN after treatment with SGF and SIF at different time points (0, 2, 4, and 8 h). Scale bars, 50 and 20 nm. (F) TEM images of RGP-VMSN degraded in SBF. Scale bars, 100 nm. Data are presented as means ± SD (n = 3 independent experiments).

Efficient antigen delivery to APCs or mucosal inductive sites is essential for an effective immune responses [40]. The physicochemical properties of nanocarriers strongly influence their dispersion, stability, and interactions with immune cells. Contact angle measurements showed a decrease from 16.2° for VMSN to 12.5° after RGP grafting (Fig. 2A). BCA analysis showed an average loading content of 163.5 μg/mg and a loading efficiency of 71.4%. After OVA loading, pore size, pore volume, and specific surface area significantly decreased (Fig. S1B–F), indicating effective antigen loading and pore occupation.

Compared with VMSN, RGP-VMSN showed slightly lower cumulative OVA release after 24 h, possibly due to the RGP coating (Fig. 2B), suggesting reduced premature antigen leakage [41]. Far-UV circular dichroism spectra showed reduced secondary-structure signals after OVA loading, likely due to interactions with pore walls. In contrast, the released antigen exhibited spectral features closer to those of free OVA than those of the loaded state (Fig. 2C). ELISA analysis further showed that released OVA retained antigen reactivity comparable to that of free OVA (Fig. S2), suggesting that loading-induced conformational changes were at least partially reversible.

To evaluate structural stability under simulated gastrointestinal conditions, nanoparticles were sequentially incubated in SGF (pH 1.2, with pepsin) for 2 h and transferred to SIF (pH 6.8, with trypsin) for an additional 2, 4, or 6 h. DLS analysis showed stable hydrodynamic size and PDI after incubation in both gastric and intestinal fluids (Fig. 2D). TEM images confirmed that the spiky morphology remained intact after sequential exposure to SGF and SIF (Fig. 2E),and the RGP retention rate remained as high as 90.3% after 24 h of simulated gastrointestinal digestion (Fig. S3), indicating good structural stability.

In SBF, RGP-VMSN gradually degraded over 7 days (Fig. 2F), suggesting slow degradation under physiological conditions. These findings support the potential of RGP-VMSN as a stable carrier for intestinal antigen delivery.

### 3.3 Epithelial interaction, antigen accessibility, and DC responses in an epithelial immune co-culture system

**Fig. 3:**
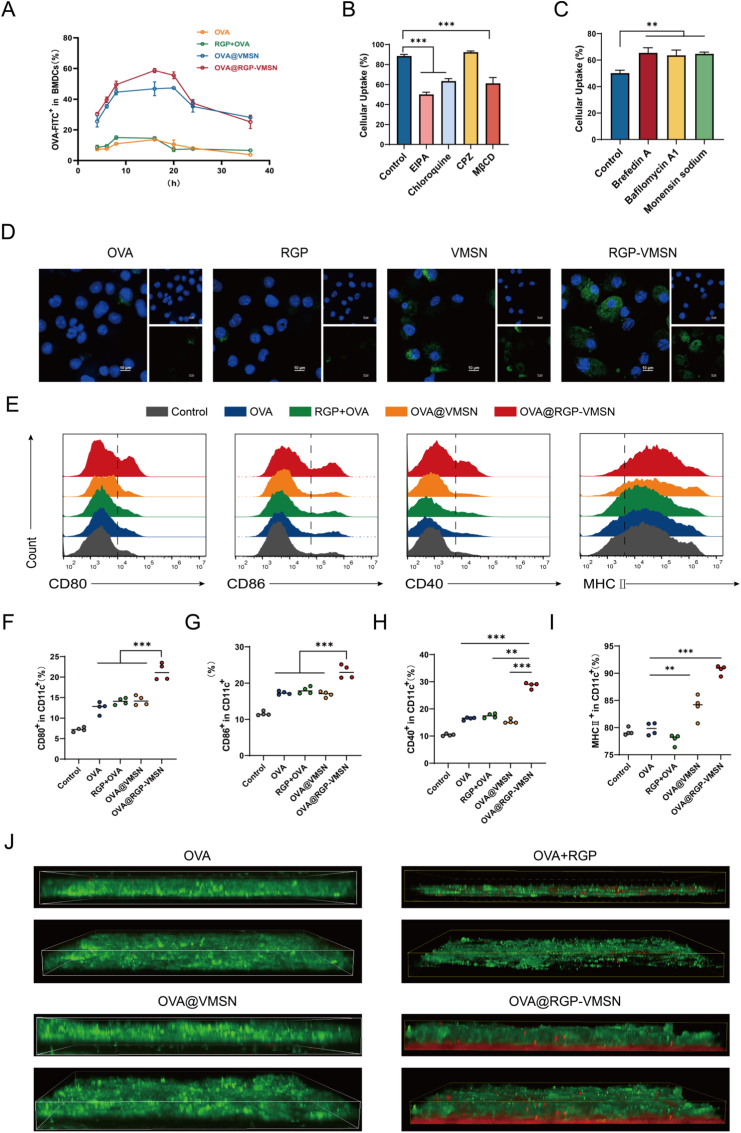
(A) Time-dependent uptake of OVA@RGP-VMSN by BMDCs in the Transwell co-culture model (n = 4 biological replicates). (B) Analysis of exocytosis pathways of OVA@RGP-VMSN in Caco-2 cells (n = 4 biological replicates). (C) Analysis of endocytosis pathways of OVA@RGP-VMSN in Caco-2 cells (n = 4 biological replicates). (D) Confocal laser scanning microscopy (CLSM) images of BMDCs treated with nanovaccines for 16 h, showing OVA-FITC (green) and nuclei stained with DAPI (blue). Scale bars, 10 μm. (E) Expression of co-stimulatory molecules (CD80, CD86, CD40, and MHC-II) on BMDCs after 16 h of co-culture with nanoparticles was analyzed using FCM. (F–I) Percentages of CD80^+^, CD86^+^, CD40^+^, and MHC-II^+^ cells of BMDCs population after 16 h of co-culture with nanoparticles. (J) Three-dimensional CLSM images of Occludin (green) and CD11c^+^ DCs (red) in the Caco-2-DC Transwell model. Data are presented as mean ± SD. Each symbol represents an individual sample (n = 4). ∗∗*P* < 0.01, and ∗∗∗*P* < 0.001 by one-way ANOVA followed by Tukey's multiple-comparison test. (For interpretation of the references to color in this figure legend, the reader is referred to the Web version of this article.)

To examine epithelial interaction and antigen-associated signals relevant to APC accessibility, we first examined its cellular uptake and intracellular trafficking in Caco-2 cells. Epithelial barrier interaction may be closely related to nanoscale architecture [42]. Inhibition studies showed that nanoparticle uptake was reduced following treatment with EIPA, chloroquine, and MβCD, but not with CPZ (Fig. 3B), suggesting involvement of macropinocytosis and cholesterol-dependent pathways rather than dominant clathrin-mediated endocytosis. Previous studies have suggested that spiky surface structures can influence local membrane curvature and mechanical interactions, potentially affecting macropinocytosis and cholesterol-sensitive uptake [43]. In addition, brefeldin A, bafilomycin A1, and monensin altered nanoparticle exocytosis (Fig. 3C), suggesting involvement of lysosomal, endoplasmic reticulum, and Golgi-associated trafficking pathways [44]. Overall, these findings suggest that OVA@RGP-VMSN uptake and intracellular trafficking may involve multiple endocytic and vesicular trafficking processes rather than a single dominant route [45].

To examine the relationship between epithelial interaction, antigen accessibility, and immune cell responses, we established a Caco-2/BMDC Transwell co-culture model. In this system, Caco-2 cells formed a polarized epithelial monolayer on the insert membrane, whereas BMDCs were cultured in the basolateral chamber to evaluate antigen delivery across the epithelial barrier.

We first quantified DC antigen uptake over time. OVA@RGP-VMSN produced a significantly higher proportion of OVA-positive BMDCs than that of other formulations (Fig. S4, Fig. 3A). The percentage of FITC-OVA^+^ BMDCs progressively increased and peaked at approximately 16 h. Confocal imaging further supported enhanced antigen uptake, showing strong intracellular fluorescence signals in BMDCs exposed to OVA@RGP-VMSN (Fig. 3D).

BMDCs in the basolateral chamber showed significantly increased expression of CD40, CD80, CD86, and MHC-II compared with that of other formulations (Fig.S5, Fig. 3E–I). These BMDCs also produced elevated levels of pro-inflammatory cytokines including IL-6, TNF-α, and IL-1β, indicating robust DC activation (Fig. S6).

Three-dimensional confocal imaging further revealed closer spatial proximity between CD11c^+^ cells and the epithelial interface in the RGP and RGP-VMSN groups (Fig. 3J). Tight-junction staining at 4h showed localized alterations in tight-junction continuity (Fig. S7). TEER measurements showed a transient decrease followed by recovery (Fig. S8), suggesting temporary epithelial barrier modulation within the measured period. In addition, CCK-8 assays showed no significant reduction in Caco-2 viability after 24 h, indicating no overt cytotoxicity under the tested conditions (Fig. S9). To further evaluate epithelial permeability, FITC-dextran probes with different molecular weights were used. OVA@RGP-VMSN increased the basolateral fluorescence signal of 4 kDa FITC-dextran, caused only a modest change in 40 kDa FITC-dextran, and did not significantly affect 70 kDa FITC-dextran passage (Fig. S10). These findings are consistent with transient and size-dependent epithelial barrier modulation that may increase antigen accessibility to mucosal immune cells, without evidence of broad nonspecific leakage of larger macromolecules. Nevertheless, potential subtle or long-term mucosal effects require further validation.

Previous studies have shown that spiky silica nanostructures exhibit stronger membrane interaction and enhanced cellular internalization than smooth particles, suggesting a topology-dependent advantage at the nano-bio interface [[46], [47], [48]]. To clarify whether the enhanced performance of OVA@RGP-VMSN was partly associated with surface topology, smooth MSNs and their RGP-modified counterparts with similar particle sizes were prepared as topology controls (Fig. S11A). In our study, FTIR spectra and thermogravimetric analysis confirmed successful RGP grafting on both smooth and spiky MSNs. Similar changes in hydrodynamic size and zeta potential after modification suggested comparable RGP surface modification between the two formulations (Fig. S11B–E). In the Caco-2/BMDC Transwell model, both MSN and RGP-MSN produced significantly lower proportions of OVA-positive BMDCs than those of the corresponding spiky formulations (Fig. S12), and confocal imaging showed a consistent trend (Fig. S13). Moreover, no obvious alteration in ZO-1 continuity or TEER values was observed in the smooth-particle groups (Figs. S8 and S14).

Collectively, these findings show that OVA@RGP-VMSN exhibits enhanced epithelial interaction and greater antigen accessibility to APCs, together with increased antigen uptake and BMDC activation.

### 3.4 In vivo intestinal distribution and antigen-associated signals in mesenteric lymphoid tissues

**Fig. 4:**
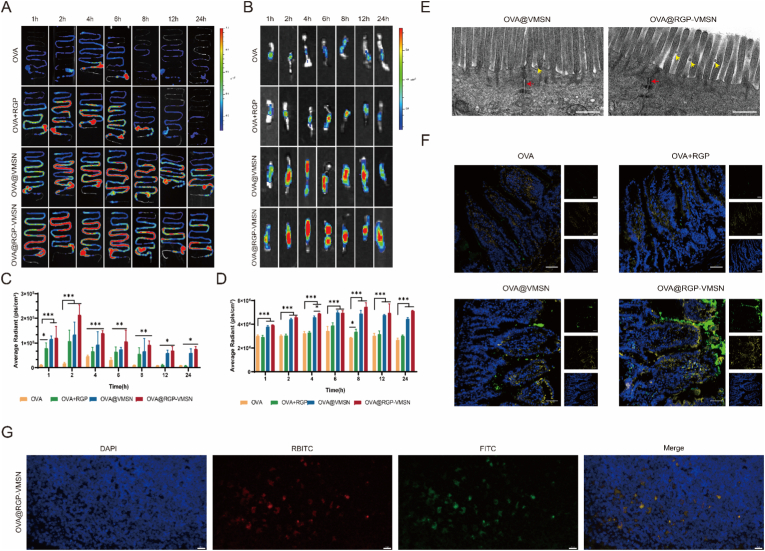
(A) *In vivo* fluorescence imaging of different OVA formulations at multiple time points after oral administration. (B) *Ex vivo* fluorescence images showing OVA-associated fluorescence signals in MLNs. (C, D) Quantitative analysis of fluorescence intensity in the (C) intestinal tract and (D) MLNs. (E) TEM images of the intestinal epithelium following oral adiministration of nanoparticles. (yellow arrows, nanoparticles; red arrows, localized widening of intercellular spaces). Scale bars, 500 nm. (F) Spatial proximity of OVA-associated signals with CD11c^+^ cells in the intestinal mucosa analyzed using fluorescence imaging (Cy5, yellow; OVA, green; DAPI, blue). Scale bars, 50 μm. (G) Adjacent FITC (green) and RBITC (red) signals in MLN tissue sections after oral FITC-OVA-loaded RBITC-labeled RGP-VMSN administration. Scale bars, 20 μm. Data are presented as means ± SD (n = 3 mice per group). ∗*P* < 0.05, ∗∗*P* < 0.01, and ∗∗∗*P* < 0.001 by one-way ANOVA followed by Tukey's multiple-comparison test. (For interpretation of the references to color in this figure legend, the reader is referred to the Web version of this article.)

To evaluate the *in vivo* behavior of the nanovaccine after oral administration, biodistribution and intestinal imaging studies were performed using Cy5.5-labeled OVA. Real-time imaging showed stronger and more sustained intestinal OVA-associated fluorescence signals in the OVA@RGP-VMSN group than in the free OVA and OVA@VMSN groups (Fig. 4A and C). Increased OVA-associated fluorescence signals were also observed in mesenteric lymphatic tissues (Fig. 4B and D). At 24 h post-administration, *ex vivo* imaging detected fluorescence signals in the lungs of both the VMSN and RGP-VMSN groups (Fig. S15).

Bio-TEM imaging revealed multipoint attachment of RGP-VMSN to the intestinal microvilli surface (Fig. 4E, yellow arrows). Localized widening of the intercellular junctional regions was observed in selected areas (Fig. 4E, red, green, and blue arrows) without obvious disruption of epithelial architecture. These findings were consistent with the epithelial alterations observed *in vitro*. Immunofluorescence staining further showed distinct antigen-associated fluorescence patterns among groups. Free OVA displayed weak and scattered signals, whereas OVA + RGP and OVA@VMSN showed enhanced antigen signals mainly along the villus surface. Notably, OVA@RGP-VMSN showed the strongest mucosal OVA-associated fluorescence and closer spatial proximity to CD11c^+^ cells. Antigen-associated signals were also detected in deeper villus regions, suggesting enhanced antigen accessibility to mucosal immune cells (Fig. 4F).

FCM analysis showed increased proportions of CCR7^+^CD103^+^CD11c^+^ cells in Peyer's patches (PPs) and the intestinal lamina propria following OVA@RGP-VMSN administration (Fig. S16). In addition, fluorescence imaging of MLNs showed adjacent FITC- and RBITC-associated signals after oral administration of FITC-OVA-loaded RBITC-labeled RGP-VMSN (Fig. 4G).

Collectively, OVA@RGP-VMSN showed enhanced intestinal retention and epithelial interaction, together with increased OVA-associated signals in the intestinal mucosa and mesenteric lymphoid tissues.

### 3.5 Induction of DC activation, Tfh responses, and GC formation

**Fig. 5:**
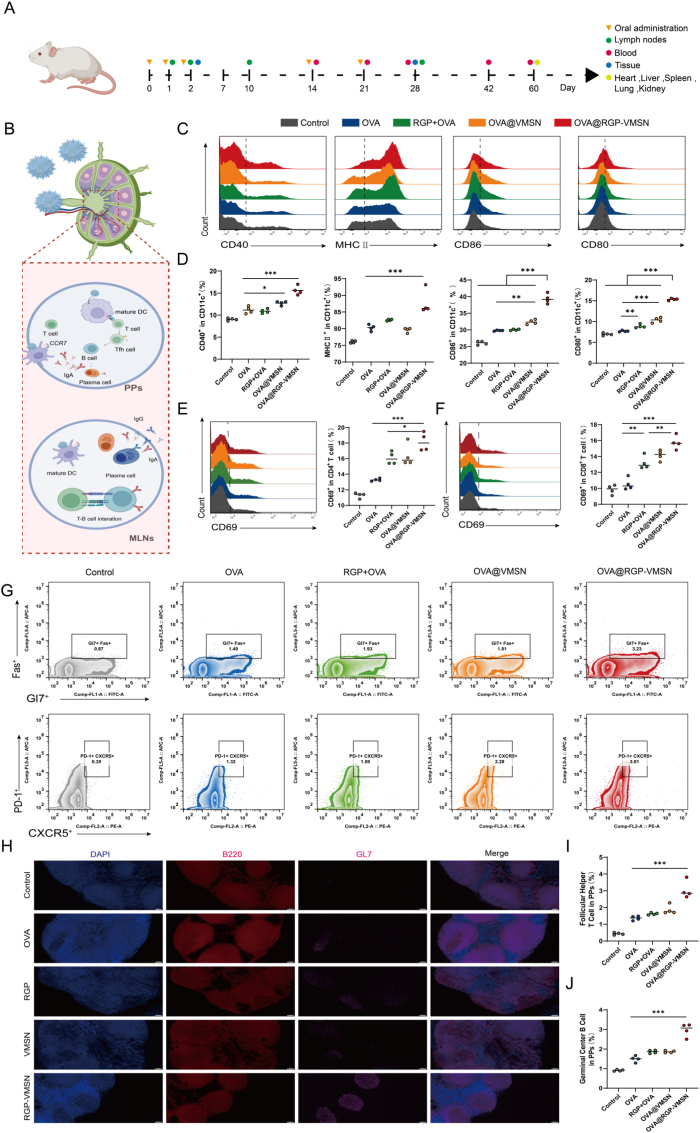
(A) Oral immunization schedule and sample collection. (B) Schematic summary of epithelial interaction, DC responses, and mucosal immune changes after oral immunization. (C, D) Representative FCM histograms and quantification of CD40, MHC II, CD86, and CD80 on CD11c^+^ DCs.(E, F) Representative FCM histograms and quantification of CD69 expression on CD4^+^ and CD8^+^ T cells. (G) FCM plots of Tfh (CXCR5^+^PD-1^+^) and GC B cells (FAS^+^GL7^+^) in PPs. (H)Immunofluorescence visualization of GC structures (B220, red; GL7, magenta; DAPI, blue) in PPs. Scale bars, 100 μm. (I, J) Quantification of Tfh cells and GC B cells in PPs. Data are presented as means ± SD. Each symbol represents an individual sample (n = 4 mice per group). ∗*P* < 0.05, ∗∗*P* < 0.01, and ∗∗∗*P* < 0.001 by one-way ANOVA followed by Tukey's multiple-comparison test. (For interpretation of the references to color in this figure legend, the reader is referred to the Web version of this article.)

The protective efficacy of oral vaccines depends on the effective activation of the gut mucosal immune system [49]. To evaluate the mucosal immune responses induced by oral OVA@RGP-VMSN, female BALB/c mice were immunized according to the schedule shown in Fig. 5A. Fig. 5B presents a Proposed working model of associated mucosal immune responses. At 12 h after oral administration, increased OVA^+^CD11C^+^ signals were detected in MLNs in the OVA@RGP-VMSN group those of the control, VMSN, and OVA groups (Fig. S17). Following oral vaccination, CD11c^+^ DCs in MLNs exhibited a mature phenotype at 24h, characterized by coordinated upregulation of CD80, CD86, CD40, and MHC-II expression (Fig.S18, Fig. 5C and D), indicating activation of APCs within the gut mucosal immune system. Early T-cell activation was also detected within 48 h after immunization (Fig. S19, Fig. 5E and F).

Further analysis showed an increased in CCR7^+^CD103^+^CD11c^+^ DC populations in PPs, the lamina propria, and MLNs at 24h (Figs. S16 and S20A), a phenotype associated with migration-related or activation-associated intestinal DC subsets [50]. At 48 h, CCR7^+^CD103^+^CD11c^+^ DC populations in these intestinal immune compartments declined compared with those at 24 h, but remained higher in the OVA@RGP-VMSN group than those in the other groups (Fig. S21). In addition, CCR7^+^CD11b^+^CD11c^+^ DCs were elevated in PPs, the lamina propria, and MLNs at 24h (Fig. S20B), suggesting coordinated enrichment of activation-associated DC subsets across intestinal immune compartments following oral immunization.

GC responses were detected in PPs, a key mucosal inductive site. FCM analysis revealed significant increases in FAS^+^GL7^+^ GC B cells and CXCR5^+^PD-1^+^ Tfh cells (Fig. S22, Fig. 5G–I). Immunofluorescence staining further confirmed enlarged GCs in PPs (Fig. 5H).

Elevated GC-associated markers were also detected in MLNs (Fig. S23), suggesting GC-related immune responses in draining lymphoid tissues. The concurrent GC-related responses observed in PPs and MLNs support coordinated immune activation across mucosal inductive and draining lymphoid compartments [51].

Overall, oral administration of OVA@RGP-VMSN enhanced GC-related responses at the mucosal inductive interface, particularly in PPs. These findings support coordinated immune activation across mucosal inductive sites and draining lymph tissues, although the precise causal relationships among these immune events require further investigation.

### 3.6 Mucosal IgA response and plasma cell activation

**Fig. 6:**
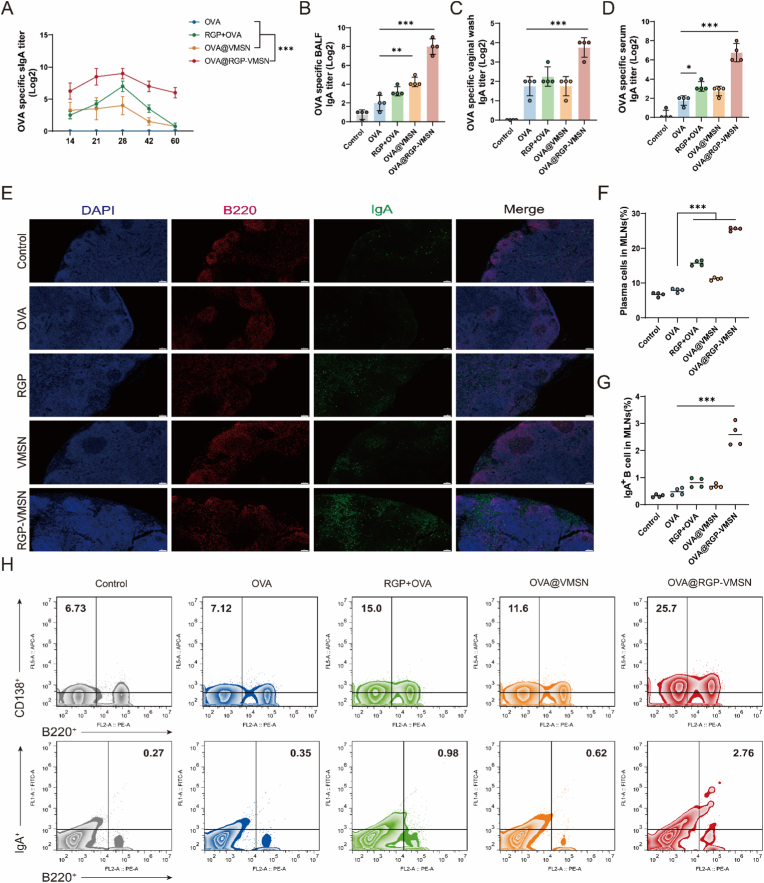
(A) OVA-specific SIgA titers in intestinal lavage fluid after oral immunization. (B–D) OVA-specific IgA titers in intestinal lavage fluid, serum, and respiratory secretions on day 28. (E) Immunofluorescence staining of MLNs showing IgA^+^ regions (green) and B220^+^ B cells (red), with nuclei stained using DAPI (blue). Scale bars, 100 μm. (F-H) Representative FCM plots and quantification of IgA^+^ B and plasma cells in MLNs. Data are presented as means ± SD. Each symbol represents an individual sample (n = 4 mice per group). For antibody time-course data, ∗∗∗*P* < 0.001 was determined by two-way ANOVA followed by Tukey's multiple-comparison test. For comparisons among multiple groups at a single time point, ∗*P* < 0.05, ∗∗*P* < 0.01, and ∗∗∗*P* < 0.001 were determined by one-way ANOVA followed by Tukey's multiple-comparison test. (For interpretation of the references to color in this figure legend, the reader is referred to the Web version of this article.)

To assess humoral immune responses, blood samples were collected on days 14, 21, 28, 42, and 60 after immunization for antibody analysis. In addition, MLNs were additionally harvested on day 28 to evaluate IgA^+^ B cells and plasma cells using FCM. OVA@RGP-VMSN yielded significantly higher antigen specific SIgA titers than those of the control, VMSN, and OVA groups (Fig. 6A), indicating strong intestinal SIgA response after booster immunization. Enhanced antibody responses were also detected in distant mucosal sites, including BALF, vaginal wash samples and serum (Fig. 6B–D). Immunofluorescence staining showed expanded IgA-positive regions in the MLNs of the OVA@RGP-VMSN group (Fig. 6E). FCM analysis further demonstrated increased plasma-cell populations (Fig. S24, Fig. 6F–H) and higher frequencies of IgA^+^ B cells (Fig. S25,Fig. 6H–G), suggesting active B-cell differentiation and antibody production.

These findings indicate that OVA@RGP-VMSN promotes coordinated mucosal and systemic humoral responses following oral immunization.

### 3.7 Long-term IgG response and T-cell effector function activation

**Fig. 7:**
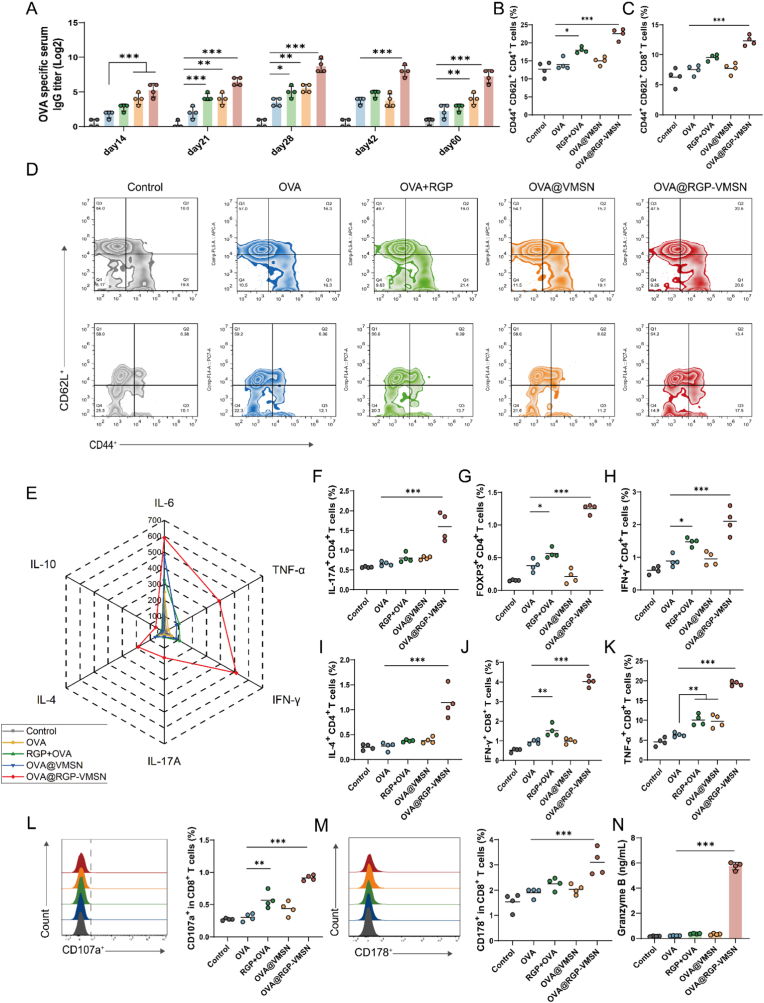
(A) OVA-specific IgG titers in serum after oral immunization. (B-D) Quantification and representative FCM plots of CD44^+^CD62L^+^ CD4^+^ and CD44^+^CD62L^+^ CD8^+^ T-cell frequencies using FCM. (E) Cytokine radar plot of IL-6, TNF-α, IFN-γ, IL-17A, IL-10, and IL-4. (F–K) Frequencies of IL-17A^+^CD4^+^, Foxp3^+^ CD4^+^, IFN-γ^+^CD4^+^, IFN-γ^+^CD8^+^, IL-4^+^CD4^+^, and TNF-α^+^CD8^+^ T cells. (L, M) FCM plots and quantification of CD107a^+^ and CD178^+^ subsets of CD8^+^ T cells. (N)Granzyme B secretion levels. Data are presented as mean ± SD. Each symbol represents an individual sample (n = 4 mice per group). ∗*P* < 0.05, ∗∗*P* < 0.01, and ∗∗∗*P* < 0.001 by one-way ANOVA followed by Tukey's multiple-comparison test.

Long-term immune responses induced by oral RGP-VMSN were evaluated by monitoring serum IgG levels over time. OVA@RGP-VMSN consistently induced significantly higher IgG levels than those of the control, VMSN, and OVA groups, at different time points (Fig. 7A). The IgG subclass analysis further showed increased IgG2b, IgG2a, and IgG1 responses (Fig. S26).

Although antibody titers provide a direct readout of humoral immunity, they do not fully capture the breadth, durability, or functional quality of vaccine-induced immune responses. Therefore, to obtain a more comprehensive and objective evaluation of the long-term immune response induced by oral RGP-VMSN, we further analyzed memory T-cell populations, cytokine production, and effector-function-associated T-cell markers. Memory T cells are characterized by long-term antigen-specific responsiveness and immunological recall capacity [52]. In this study, OVA@RGP-VMSN significantly increased the frequencies of CD4^+^CD44^+^CD62L^+^ and CD8^+^CD44^+^CD62L^+^ central memory T cells (Fig. S27–28, Fig. 7B–D). This increase was accompanied by elevated production of IL-6, TNF-α, IFN-γ, IL-17A, IL-10, and IL-4 (Fig. 7E), indicating enhanced T-cell activation. FCM analysis further demonstrated increased proportions of IL-17A^+^ CD4^+^, IFN-γ^+^ CD4^+^, IFN-γ^+^ CD8^+^, IL-4^+^ CD4^+^, and TNF-α^+^ CD8^+^ T cells (Fig. S29, Fig. 7F–K). These findings indicate induction of a mixed Th1/Th2/Th17 response rather than a single polarized T-helper profile, suggesting broad and coordinated immune activation [53]. The concurrent increase in Treg cells further suggests a tightly regulated immune activation that may help maintain immune homeostasis and reduce the risk of immunopathology (Fig. 7G). Elevated SIgA levels were observed in intestinal and distal mucosal sites and were accompanied by increased serum IgG responses. In addition, increased frequencies of degranulation-associated (CD107a^+^) and cytotoxic marker-positive (CD178^+^) CD8^+^ T cells were detected (Fig. 7L–N).

Together, these findings indicate that oral RGP-VMSN promotes broad adaptive immune activation and induces a sustained yet balanced immune response, highlighting its potential as a promising mucosal vaccine platform [54].

### 3.8 Enhanced protective immunity against Salmonella challenge

**Fig. 8:**
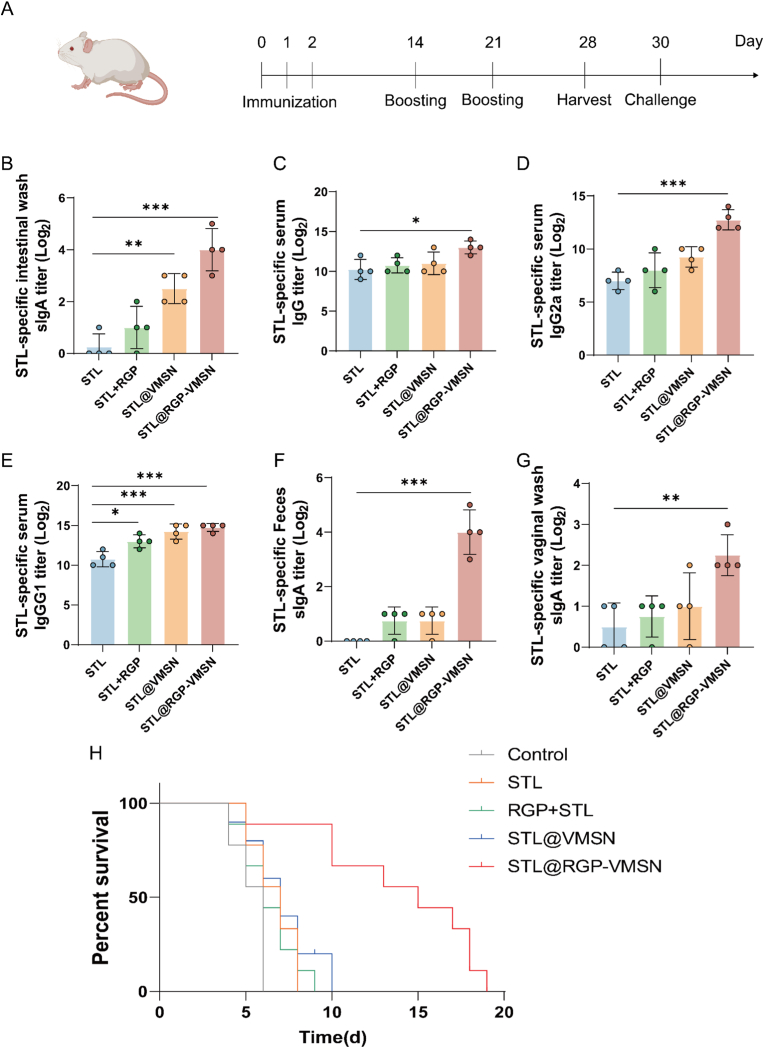
(A) Immunization and challenge schedule. (B–E) STL-specific SIgA and IgG titers in intestinal lavage fluid and serum before challenge (n = 4 mice per group). (F, G) STL-specific SIgA titers in fecal and vaginal lavage samples (n = 4 mice per group). (H) Survival curves of mice after oral challenge with *S*. Typhimurium (n = 9 mice per group). For antibody and SIgA analyses, data are presented as means ± SD, and each symbol represents one mouse (n = 4 mice per group). ∗*P* < 0.05, ∗∗*P* < 0.01, and ∗∗∗*P* < 0.001 by one-way ANOVA followed by Tukey's multiple-comparison test. Survival analysis was performed using the log-rank (Mantel-Cox) test (n = 9 mice per group).

After characterizing antigen-associated distribution and immune responses using OVA as a model antigen, we further evaluated whether the same RGP-VMSN carrier design could support protective immunity in a pathogen-related antigen setting. The physicochemical characterization and antigen release profiles of STL@RGP-VMSN are shown in Table S3 and Fig. S30, respectively. A Salmonella challenge model was subsequently established (Fig. 8A).

Mice immunized with the STL@RGP-VMSN showed significantly higher pre-challenge serum IgG and subclass levels than those of the control, VMSN, and STL groups (Fig. 8B–E). Local mucosal immune responses also showed significantly increased SIgA titers in fecal and vaginal lavage samples (Fig. 8F and G). Following oral challenge, the STL@RGP-VMSN group exhibited delayed mortality compared with that of the other groups (Fig. 8H). Together, these findings suggest that the RGP-VMSN platform supports both systemic and mucosal immune responses in this STL-based pathogen-related model and improves protection under the present experimental conditions. It should be noted that STL is a complex bacterial lysate antigen, and the magnitude and durability of antibody responses may be influenced by antigen composition, epitope abundance, and antigen accessibility. Therefore, further optimization of antigen formulation and immunization regimens, together with additional infection-related endpoints, will be needed to more comprehensively evaluate long-term protective efficacy.

### 3.9 Safety analysis

To evaluate biocompatibility after oral administration, major organs, including the heart, liver, spleen, lungs and kidneys, were examined with H&E staining on day 60 after immunization. No obvious histopathological abnormalities were observed in the RGP, VMSN, OVA@VMSN, and OVA@RGP-VMSN (Fig. S31A). Myocardial fibers remained well aligned, hepatic lobular architecture was preserved, and the white pulp and red pulp structures appeared normal. Pulmonary alveolar structures remained intact, without signs of edema or inflammatory-cell infiltration. Additionally, renal glomeruli and tubules showed no obvious swelling or damage. Serum biochemical markers associated with hepatic and renal function, including ALT, AST, ALP, BUN, and LDH, showed no significant abnormalities with those of the control group (Fig. S31B).

To further assess long-term intestinal safety, intestinal tissues collected at the same time point were examined using H&E staining at later time points. No evident inflammatory infiltration, epithelial disruption, or other obvious pathological abnormalities were observed in the OVA@RGP-VMSN group under the present experimental conditions. Consistently, long-term ZO-1 staining showed no obvious alteration in epithelial tight-junction distribution compared with that of the control group (Fig. S32), suggesting no overt persistent barrier abnormalities were observed at the examined time point.

Overall, these findings support the favorable systemic biocompatibility of both the silica nanocarrier and the RGP coating. Under the dosing conditions used in this study, oral administration of OVA@RGP-VMSN did not cause evident systemic toxicity or persistent intestinal injury.

## 4 Conclusions

In summary, we developed an oral nanovaccine platform based on spiky MSNs functionalized with RGP. This integrated design showed enhanced epithelial interaction and antigen accessibility within the intestinal mucosal environment, together with increased immune activation.

Using OVA as a model antigen, the platform induced coordinated mucosal and systemic immune responses, including SIgA production; serum IgG responses; and activation of Tfh cells, GC B cells, and effector T-cell subsets, supporting its ability to promote intestinal antigen-specific immunity. In addition, in a separate STL-based pathogen-related evaluation, the same carrier strategy showed protective potential in a Salmonella challenge model. Moreover, future studies should further optimize immunization regimens, including booster strategies, and incorporate infection-related endpoints to evaluate the durability and mechanism of long-term protection.

Overall, these findings support the potential of the RGP-VMSN platform for oral antigen delivery and mucosal immune induction in mice under the antigen systems evaluated in this study.

## CRediT authorship contribution statement

**Lin Yu:** Conceptualization, Data curation, Investigation, Methodology, Writing – original draft. **Xiaofeng Shi:** Investigation, Writing – review & editing. **Yaming Yu:** Methodology, Writing – review & editing. **Gaofeng Cai:** Investigation, Writing – review & editing. **Xuping Zhao:** Investigation. **Fangzhu Lin:** Investigation, Writing – review & editing. **Xuanqi Lu:** Methodology, Writing – review & editing. **Jie Qiao:** Writing – review & editing. **Yushan Fang:** Investigation, Writing – review & editing. **Kexin Fang:** Methodology, Supervision, Writing – review & editing. **Yang Yang:** Methodology, Writing – review & editing. **Deyun Wang:** Funding acquisition.

## Funding

This project was supported by the National Natural Science Foundation of China (Grant No. 32373053), the National Key Research and Development Program (2022YFD1801103). We are grateful to all other members of staff at the Institute of Traditional Chinese Veterinary Medicine of Nanjing Agricultural University who assisted with this study.

## Declaration of competing interest

The authors declare the following financial interests/personal relationships which may be considered as potential competing interests: Deyun Wang reports financial support was provided by the National Key Research and Development Program. Deyun Wang reports financial support was provided by the National Natural Science Foundation of China. If there are other authors, they declare that they have no known competing financial interests or personal relationships that could have appeared to influence the work reported in this paper.

## Data Availability

Data will be made available on request.
