# Supplementary material for: Spiky mesoporous silica-based nanovaccines enhance mucosal immunity with improved intestinal retention and antigen accessibility
Source: Mater Today Bio. 2026 Jul 1;39:103391. doi: 10.1016/j.mtbio.2026.103391 (PMC13351886; doi:10.1016/j.mtbio.2026.103391)
Supplement: Multimedia component 1 [file mmc1.docx]

Supporting Information

**Spiky mesoporous silica-based nanovaccines enhance mucosal immunity with improved intestinal retention and antigen accessibility**

Lin Yu^a^, Xiaofeng Shi^a^, Yaming Yu^a^, Gaofeng Cai^b^, Xuping Zhao^a^, Fangzhu Lin^a^, Xuanqi Lu^a^, Jie Qiao^a^, Yushan Fang^a^, Kexin Fang^a^, Yang Yang^a^, ***Deyun Wang****^a^****^#^***

a: Institute of Traditional Chinese Veterinary Medicine, College of Veterinary Medicine, Nanjing Agricultural University, Nanjing 210095, PR China.

b: Jiangxi Provincial Key Laboratory for Animal Health, College of Animal Science and Technology, Jiangxi Agricultural University, Nanchang 330045, Jiangxi, PR China

*Corresponding author at: Institute of Traditional Chinese Veterinary Medicine, College of Veterinary Medicine, Nanjing Agricultural University, Nanjing 210095, China. E-mail address: dywang@njau.edu.cn (D. Wang).

1.***Extraction and Purification of RGP:***

**Material:** RGP was purchased from Shanxi Ciyuan Biotechnology Co. Ltd. (Batch number: CY20220306).

**Extraction Process:**

Lipophilic components were removed by refluxing *Rehmannia glutinosa* with anhydrous ethanol for 3 hours. The polysaccharide was extracted by water extraction and alcohol precipitation.The crude product of RGP was obtained by centrifuging at 5000 ×g for 10 minutes.The crude polysaccharide was then purified by removing proteins using chloroform/n-butanol (4:1). Finally, RGP was freeze-dried for further use.

**Monosaccharide Composition:**

The monosaccharide composition of RGP includes fucose, rhamnose, arabinose, galactose, glucose, xylose, mannose in a specific molar ratio: Fuc (1.05%), Rha (18.98%), Ara (28.12%), Gal (30.54%), Glc (12.60%), Xyl (3.26%), Man (5.49%).

Fourier Transform Infrared (FT-IR) Analysis: The FT-IR spectrum of RGP shows characteristic absorption bands: O-H Stretching around 3435.08 cm^−1^ (indicative of sugars).C–O Stretching around 1632.71 cm^−1^.C–O Stretching around 1077.42 cm^−1^.

**Molecular Weight Analysis:**

The molecular weight (Mw) and number molar masses (Mn) were determined using Gel Permeation Chromatography (GPC-MALLS): Mw: 145.821 kDa, Mn: 64.059 kDa

**2. *Preparation of MSN and RGP-MSN:***

Cetyltrimethylammonium bromide (CTAB, 1.0 g) was dissolved in a mixed solution containing distilled water (480 mL), absolute ethanol (120 mL), and ammonium hydroxide solution (3.5 mL) under magnetic stirring at 40 ℃. After CTAB was completely dissolved, tetraethoxysilane (TEOS, 5 mL) was added dropwise to the above solution, and the reaction mixture was stirred at 700 rpm for 10 h.

The resulting precipitate was collected by centrifugation at 8000 rpm for 10 min and washed several times with distilled water and absolute ethanol. The obtained sample was dried overnight at 55 ℃ and then calcined in a muffle furnace at 550 ℃ for 6 h to remove the CTAB template, yielding MSN.

For the preparation of RGP-MSN, MSN was first aminated with APTES. Briefly, MSN was dispersed in toluene and stirred at 80 °C, followed by the addition of APTES. After overnight reaction, the obtained MSN-NH₂ was collected by centrifugation, washed with ethanol and distilled water, and dried. Subsequently, the carboxyl groups of RGP were activated using an EDC/NHS coupling system. The activated RGP solution was added to the MSN-NH_2_ suspension and reacted to allow covalent conjugation through amide bond formation. Finally, the product was centrifuged, washed thoroughly with distilled water to remove unreacted RGP and coupling reagents, and freeze-dried to obtain RGP-MSN.

3.***The retention of RGP on RGP-VMSN after simulated gastrointestinal digestion:***

The retention of RGP on RGP-VMSN after simulated gastrointestinal digestion was evaluated using the phenol–sulfuric acid method. Briefly, RGP-VMSN was sequentially incubated in SGF (pH 1.2, containing pepsin) for 2 h and then in SIF (pH 6.8, containing trypsin) for up to 24 h. At predetermined time points, the digestion mixture was heated at 95 °C for 15 min to inactivate digestive enzymes. The samples were then centrifuged to separate nanoparticles from the digestion medium, and the supernatants were collected to determine the amount of RGP released from RGP-VMSN. The supernatants were reacted with 5% phenol and concentrated sulfuric acid, and the absorbance was measured at 490 nm. The released RGP content was calculated from a standard curve generated using anhydrous glucose.

The RGP retention rate was calculated as follows:

RGP retention rate (%) = $\frac{W_{0}-W_{r}}{W_{0}}\times100\%$

W₀ is the initial amount of grafted RGP and Wᵣ is the amount of RGP released into the digestion medium.

**Table S1 Major antibodies used for FCM.**

| **Antibody** | **Manufacturer** | **Antibody** | **Manufacturer** |  |
| --- | --- | --- | --- | --- |
| CD80-FITC | BioLegend | CD11C-PE/CY7 | BioLegend | |
| CD86-PE | BioLegend | CD40-APC | BioLegend |  |
| Foxp3-APC | Thermo Fisher Scientific | MHCⅡ-APC | BioLegend |  |
| CD69-APC | BioLegend | CD3-FITC | BioLegend |  |
| CD8-PE | BioLegend | CD8-PE/CY7 | Thermo Fisher Scientific |  |
| CD8-APC | BioLegend | CD4-PE | BioLegend |  |
| CD4-APC | BioLegend | CD4-PE/CY7 | BioLegend |  |
| CD138-APC | BioLegend | B220-PE | Thermo Fisher Scientific |  |
| CD107a-FITC | BioLegend | CD178-PE | BioLegend |  |
| IgA-FITC | BioLegend | FAS/CD95-APC | Thermo Fisher Scientific |  |
| GL7-FITC | Thermo Fisher Scientific | CD62L-FITC | BioLegend |  |
| IFN-γ-APC | Thermo Fisher Scientific | CD44-PE/Cy7 | BioLegend |  |
| TNF-α-PE | BioLegend | IL-4-PE | Thermo Fisher Scientific |  |
| PD-1-APC | Thermo Fisher Scientific | CXCR5-PE | Thermo Fisher Scientific |  |
| IL-17A-PE | Elabscience |  |  |  |

**Table S2 Specific surface areas (SBET), pore volumes (Vt) and pore diameter (PD) of VMSN, RGP-VMSN, OVA@VMSN, OVA@RGP-VMSN.**

| Sample | S_BET_ (m^2^/g) | Vt (cm^3^/g) | P_D_ (nm) |
| --- | --- | --- | --- |
| VMSN | 421.13 | 1.12 | 10.6 |
| RGP-VMSN | 128.53 | 0.62 | 7.4 |
| OVA@VMSN | 90.15 | 0.35 | 3.8 |
| OVA@RGP-VMSN | 91.11 | 0.28 | 4.1 |

**Table S3 Characteristics of the nano-vaccines.**(n = 3 independent experiments)

| **Sample** | **Size (nm)** | **PDI** | **Zeta Potential (mV)** | **STL-LE (%)** |
| --- | --- | --- | --- | --- |
| STL@VMSN | 310.1 ± 8.2 | 0.16 ± 0.020 | -15.4 ± 0.3 | 70.35 ± 0.273 |
| STL@RGP-VMSN | 317.3 ± 7.1 | 0.29 ± 0.055 | -13.9 ± 0.7 | 72.38 ± 0.548 |


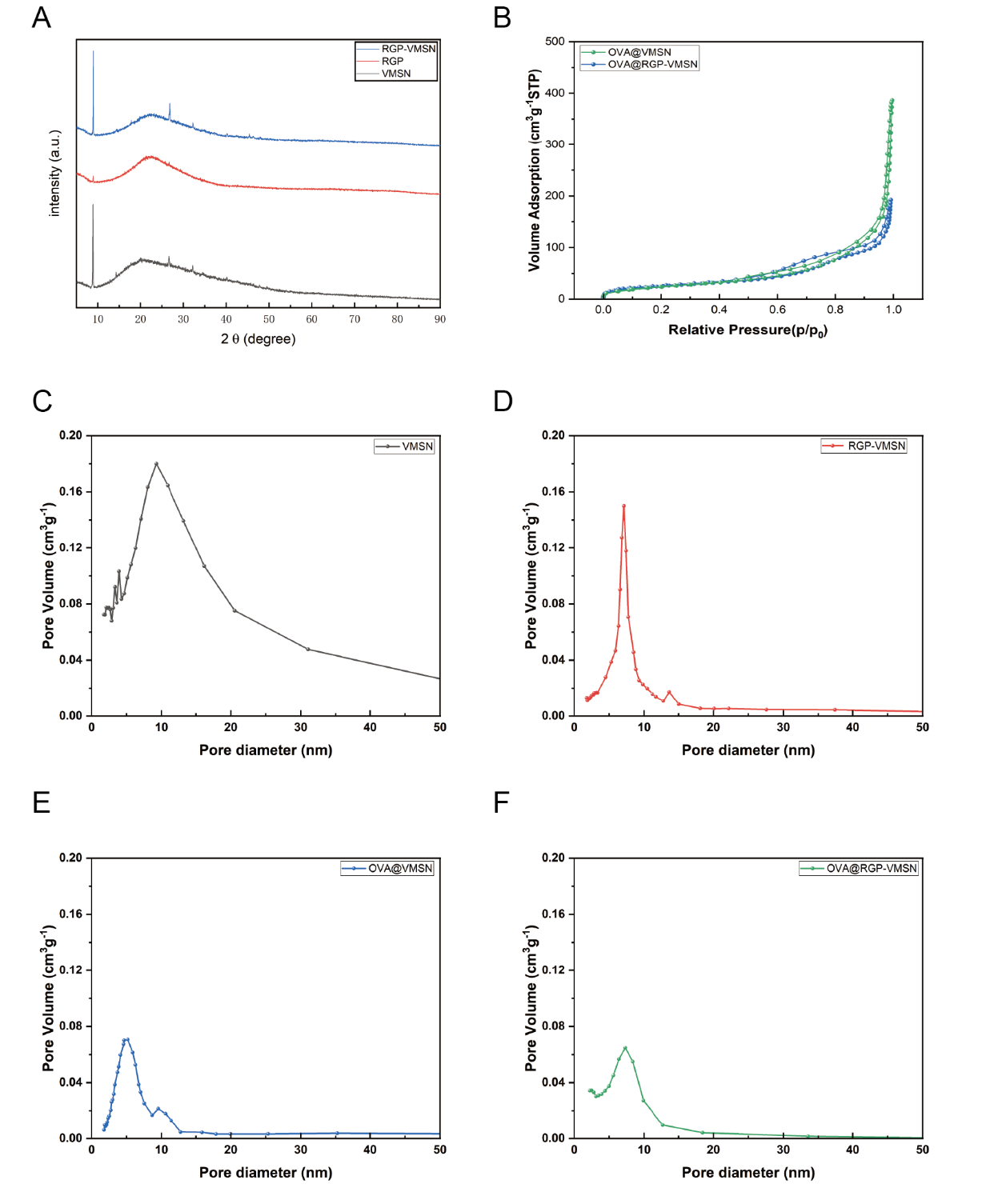


**Fig. S1.** (A) XRD patterns of VMSN, RGP-VMSN, and RGP. (B) Nitrogen adsorption–desorption isotherms and pore size distribution curves of OVA@VMSN and OVA@RGP-VMSN. (C–F) Pore size distribution profiles of VMSN, RGP-VMSN, OVA@VMSN, and OVA@RGP-VMSN. Experiment was repeated three times independently with similar results.


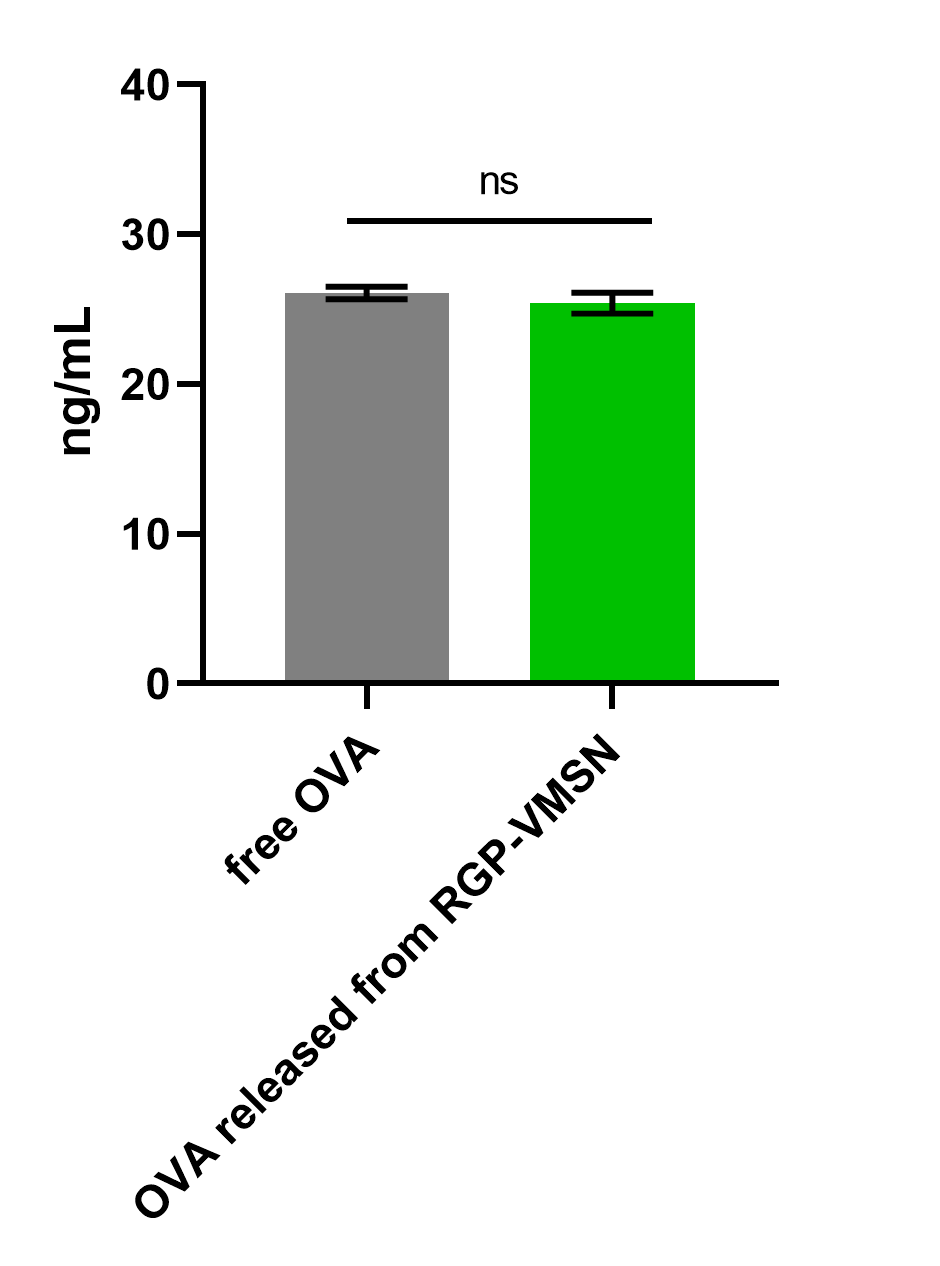


**Fig. S2.** Quantification of free OVA and OVA released from OVA@RGP-VMSN.Data are presented as mean ± SD (n = 3 independent experiments); ns, not significant by one-way ANOVA followed by Tukey’s multiple-comparison test.


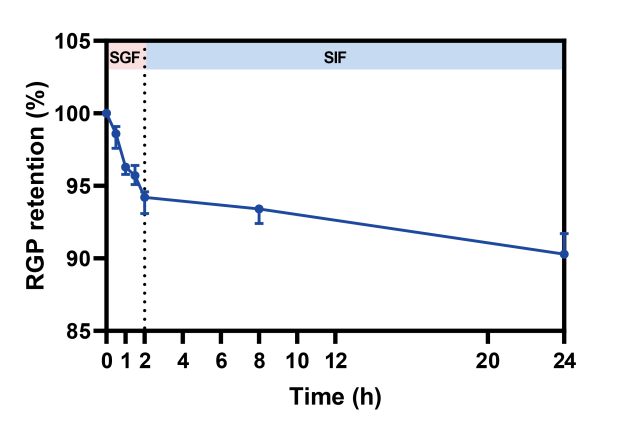


**Fig. S3.** Retention of RGP after sequential incubation in SGF (without pepsin) and SIF (without trypsin) at 37 ℃.Data are expressed as mean ± SD (n = 3 independent experiments).


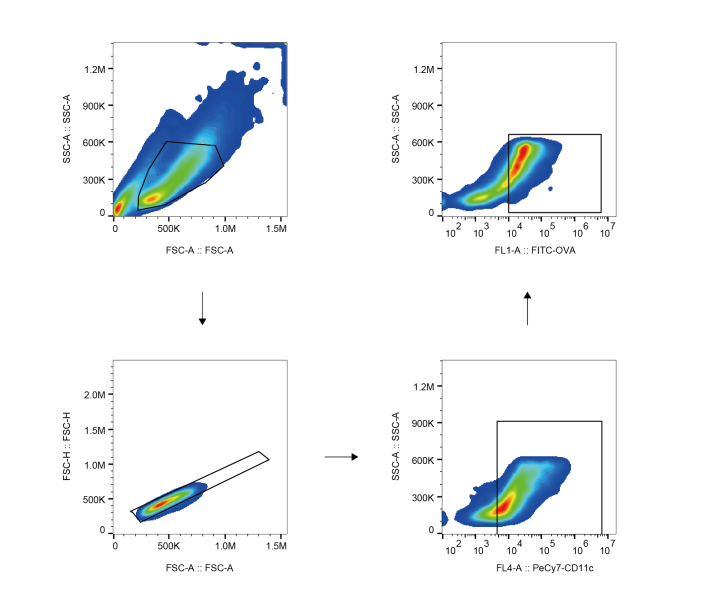


**Fig. S4.** Gate strategies for the uptake of OVA-FITC in BMDCs.


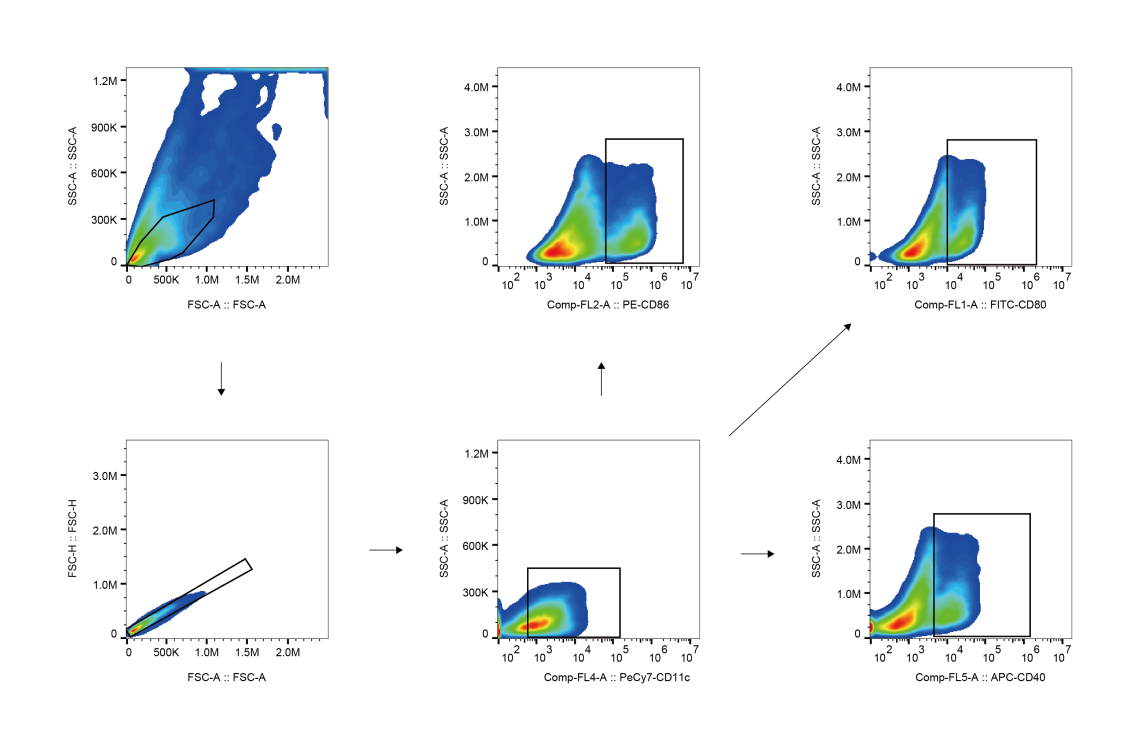


**Fig. S5.** FCM analysis of gating strategies to measure co-stimulatory molecules expression (CD40, CD80, and CD86) in BMDCs.


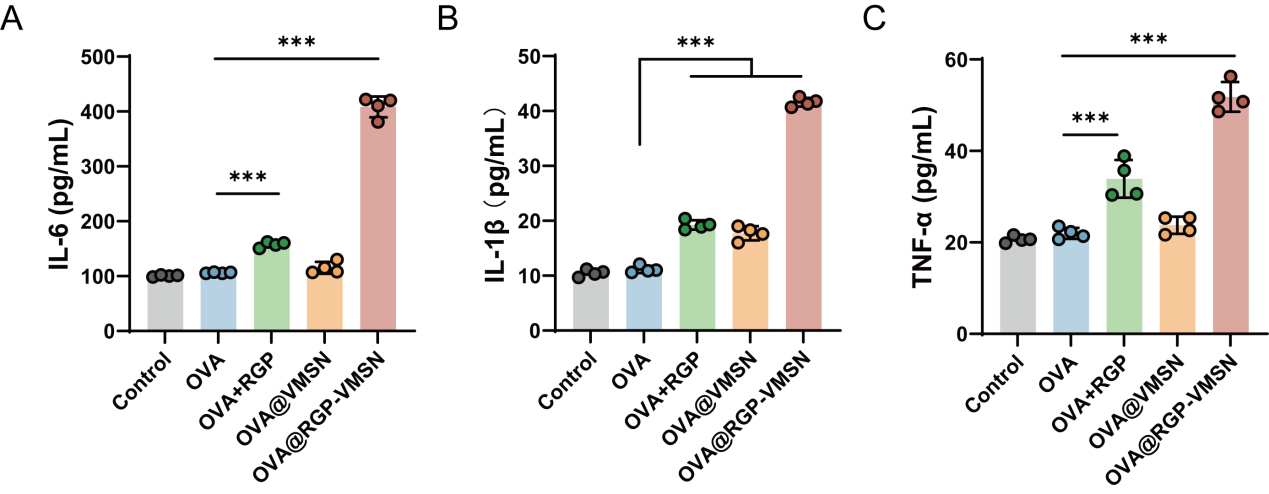


**Fig. S6.** The content of IL-6 (A), IL-1β(B), and TNF-α(C) in the supernatants of BMDCs was measured by ELISA. Data are presented as mean ± SD. Each symbol represents an individual sample (n = 4 mice per group ). ****P* < 0.001 by one-way ANOVA followed by Tukey’s multiple-comparison test.


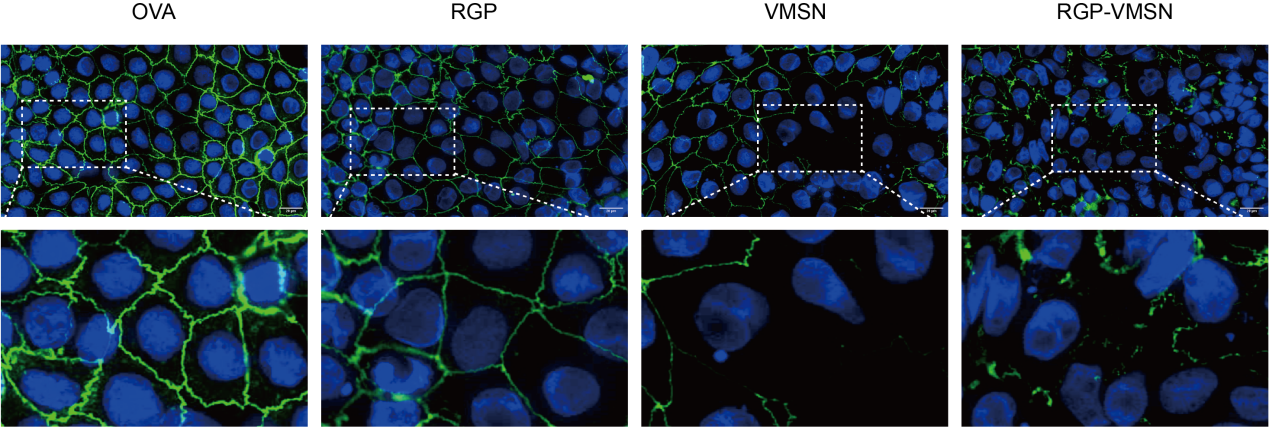


**Fig. S7.** Immunofluorescence staining of tight junction protein ZO-1 (green) in intestinal epithelial tissues . Nuclei were counterstained with DAPI (blue). Scale bar = 20 μm. All images are representative of independent biological samples (n = 3).

.
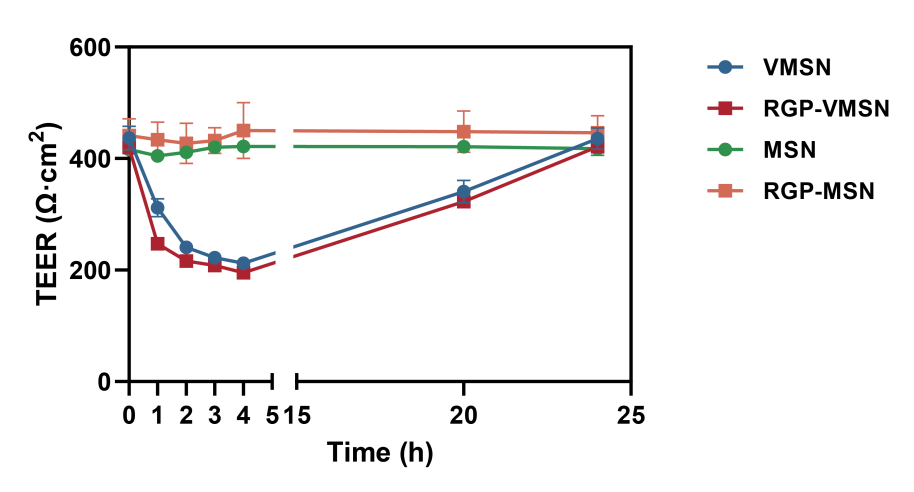


**Fig. S8**. The TEER before and after the addition of VMSN, RGP-VMSN, MSN,and RGP-MSN. Data are presented as mean ± SD. (n = 3 independent experiments)


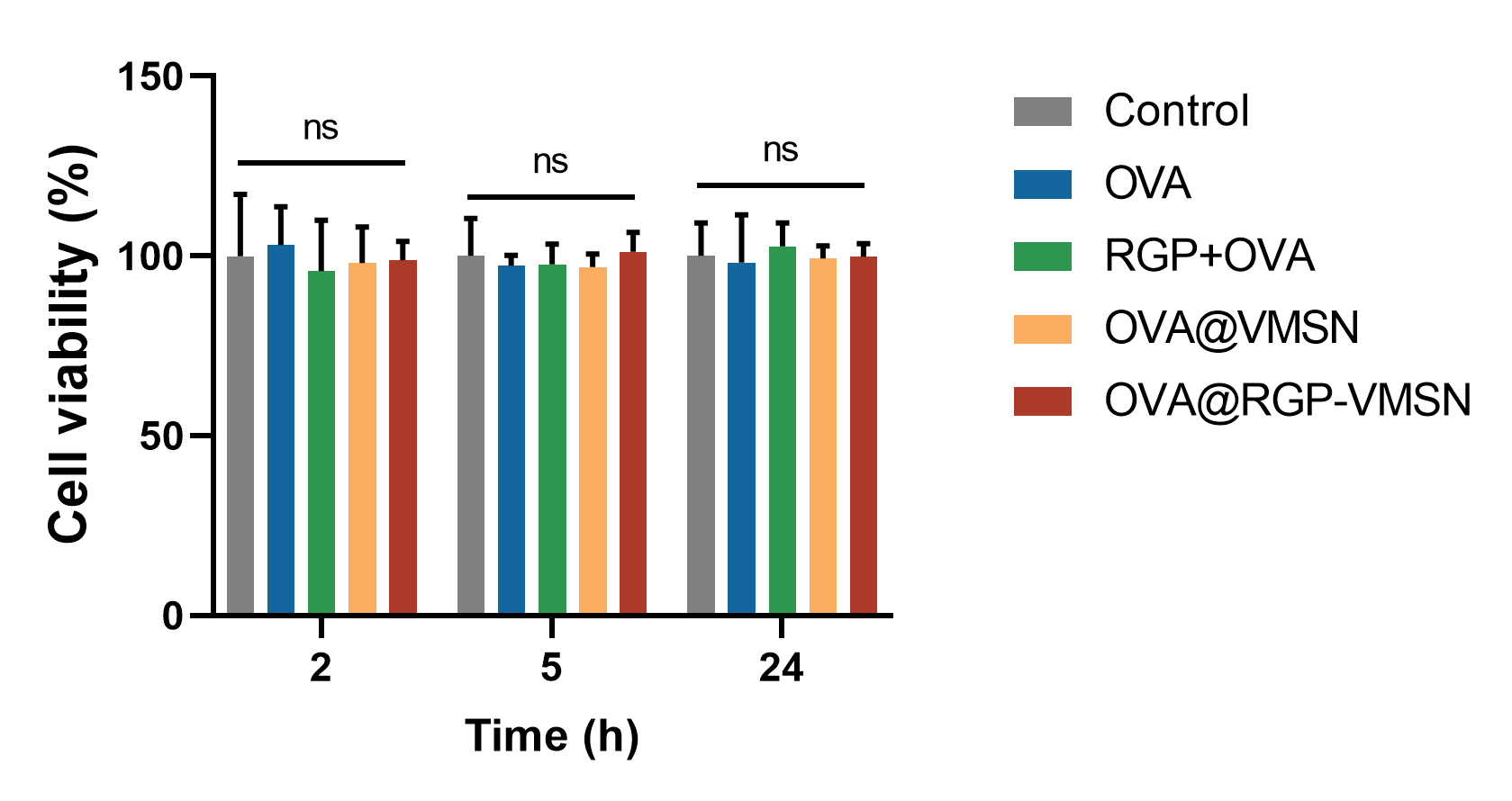


**Fig.S9.** Cell relative viability of Caco-2 after incubation with formulations. Data are presented as mean ± SD. (n = 3 independent experiments); ns, not significant by one-way ANOVA followed by Tukey’s multiple-comparison test.


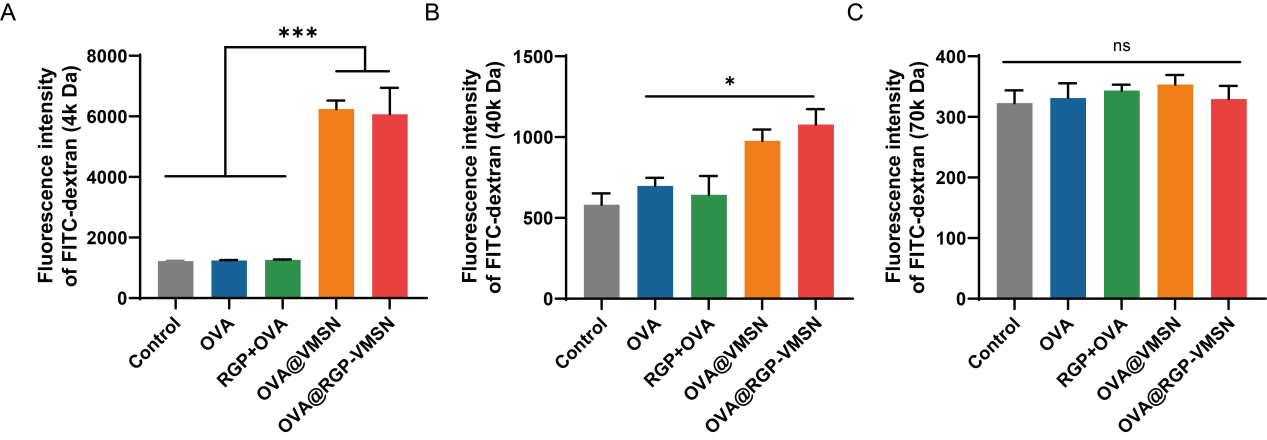


**Fig. S10.** Fluorescence intensity of FITC-dextran in the basolateral chamber was measured for (A) 4 kDa, (B) 40 kDa, (C) and 70 kDa. Data are presented as mean ± SD. (n = 3 independent experiments) **P* < 0.05, ****P* < 0.001, ns, not significant by one-way ANOVA followed by Tukey’s multiple-comparison test.


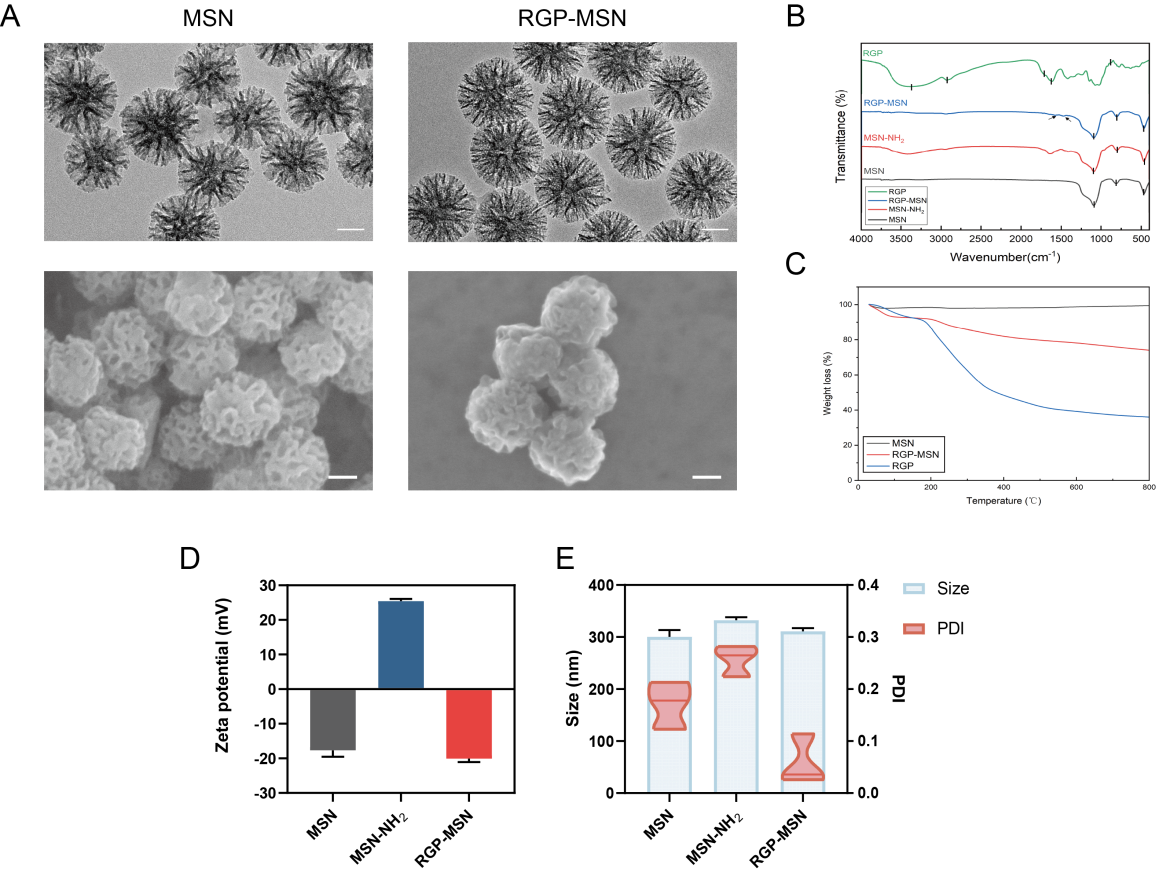


**Fig.S11.** (A) TEM images and SEM images of MSN and RGP-MSN. Scale bars, 100nm (B) FTIR spectra of MSN, MSN-NH₂, RGP-MSN and RGP. (C) TGA curves of nanoparticles. (D) Zeta potentials of nanoparticles. (E)Particle size and PDI of nanoparticles.Data are presented as mean ± SD. (n = 3 independent experiments)


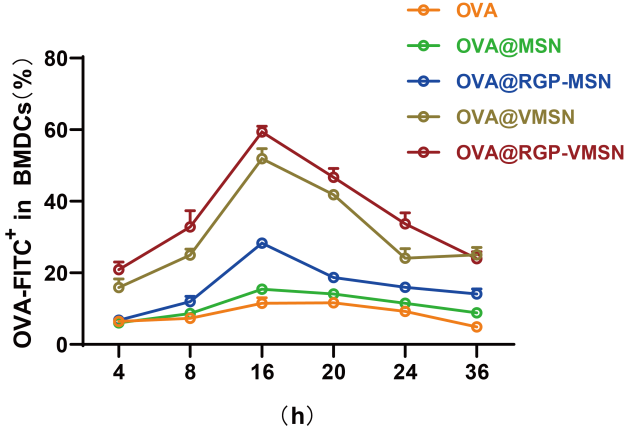


**Fig. S12.** Time-dependent uptake of nanoparticles by BMDCs in the Transwell co-culture model. Data are presented as mean ± SD. (n = 3 independent experiments)


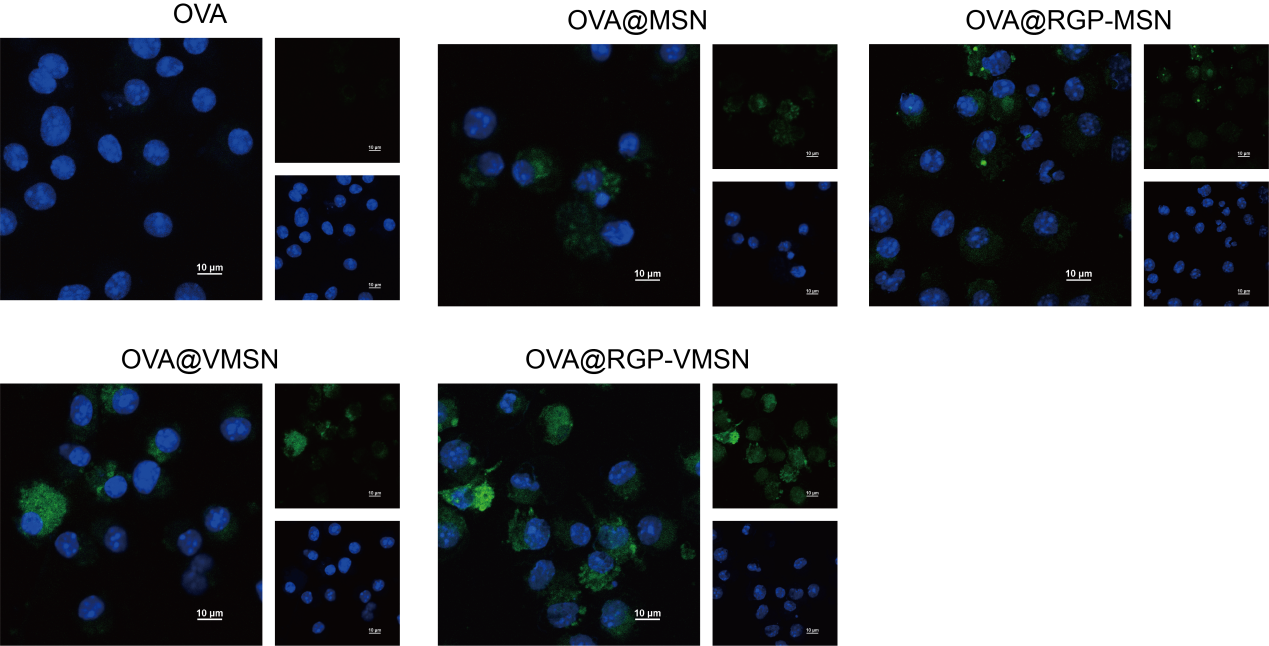


**Fig. S13.**  CLSM images of nanovaccine-treated BMDCs after 16 hours with OVA-FITC in green, nuclei in blue (DAPI). Scale bars, 10μm. All images are representative of independent biological samples (n = 3).


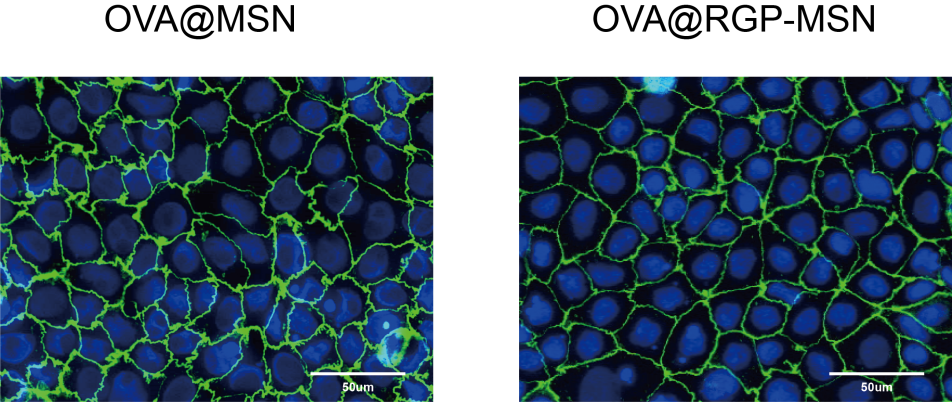


**Fig. S14.** Immunofluorescence staining of tight junction protein ZO-1 (green) in intestinal epithelial tissues . Nuclei were counterstained with DAPI (blue). Scale bar = 20 μm. All images are representative of independent biological samples (n = 3)


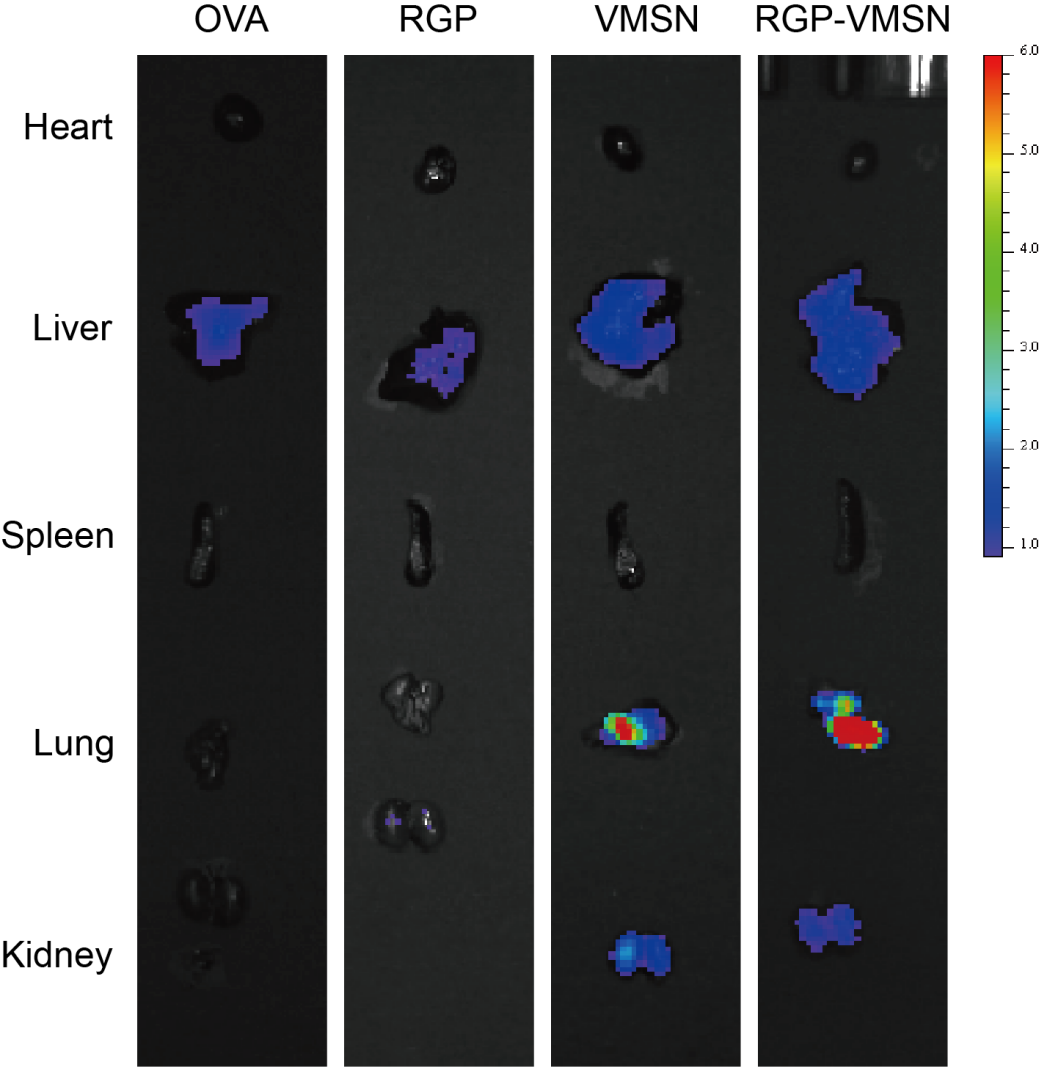


**Fig. S15.** *In vivo* fluorescence imaging of major organs (Heart, Liver, Spleen, Lung, and Kidney) at 24 h post oral administration of different OVA formulations. All images are representative of n = 3 samples per group.


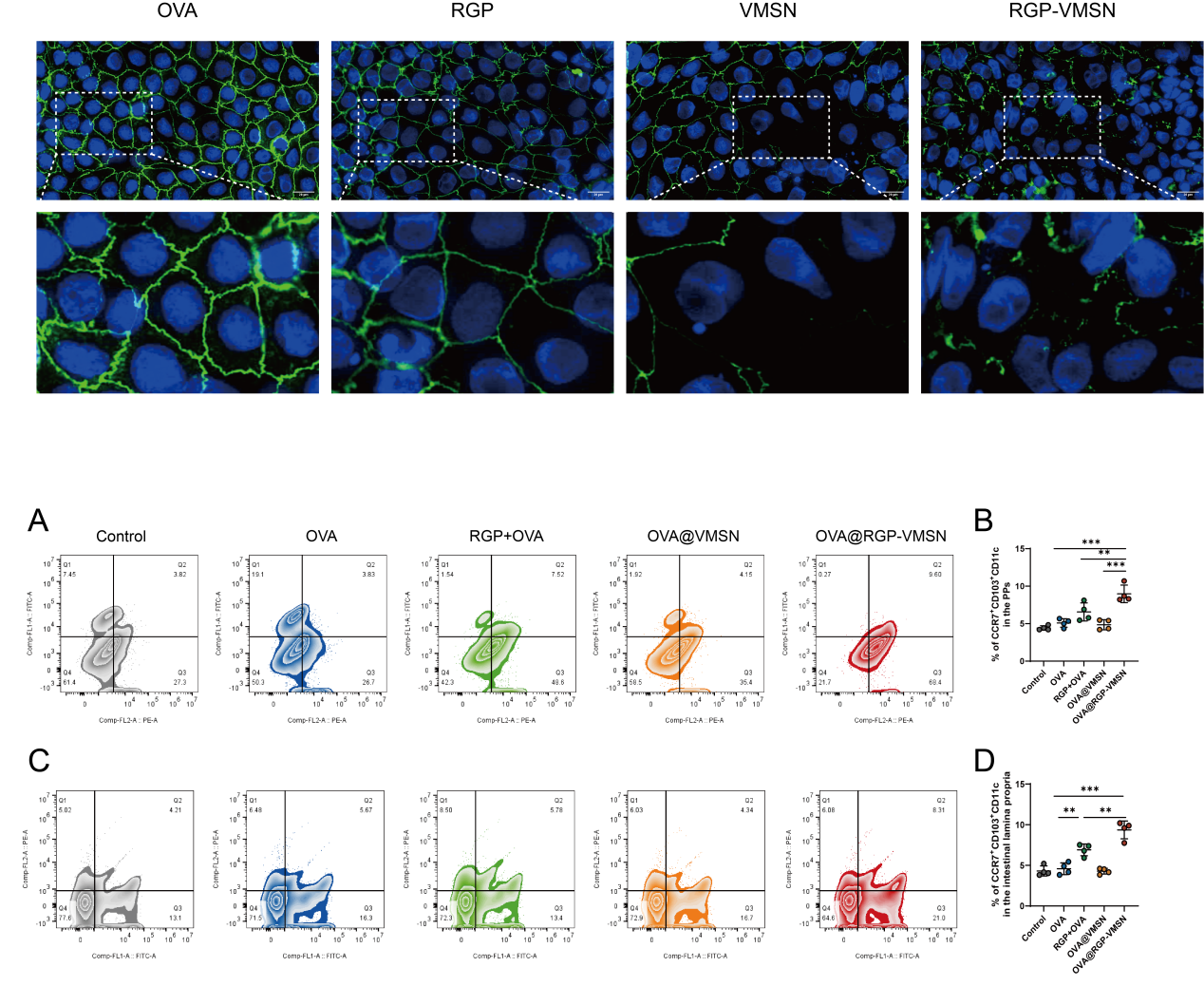


**Fig. S16.** At 24 h after oral administration, (A, B) Flow cytometry plots and quantitative analysis of CCR7^+^CD103^+^CD11c^+^ cells in PPs patches . (C, D) Flow cytometry plots and quantification of CCR7^+^CD103^+^CD11c^+^ cells in the intestinal lamina propria. Each symbol represents an individual sample (n = 4 mice per group ). Data are expressed as means ± SD. ****P* < 0.001, ***P* < 0.01 by one-way ANOVA followed by Tukey’s multiple-comparison test.


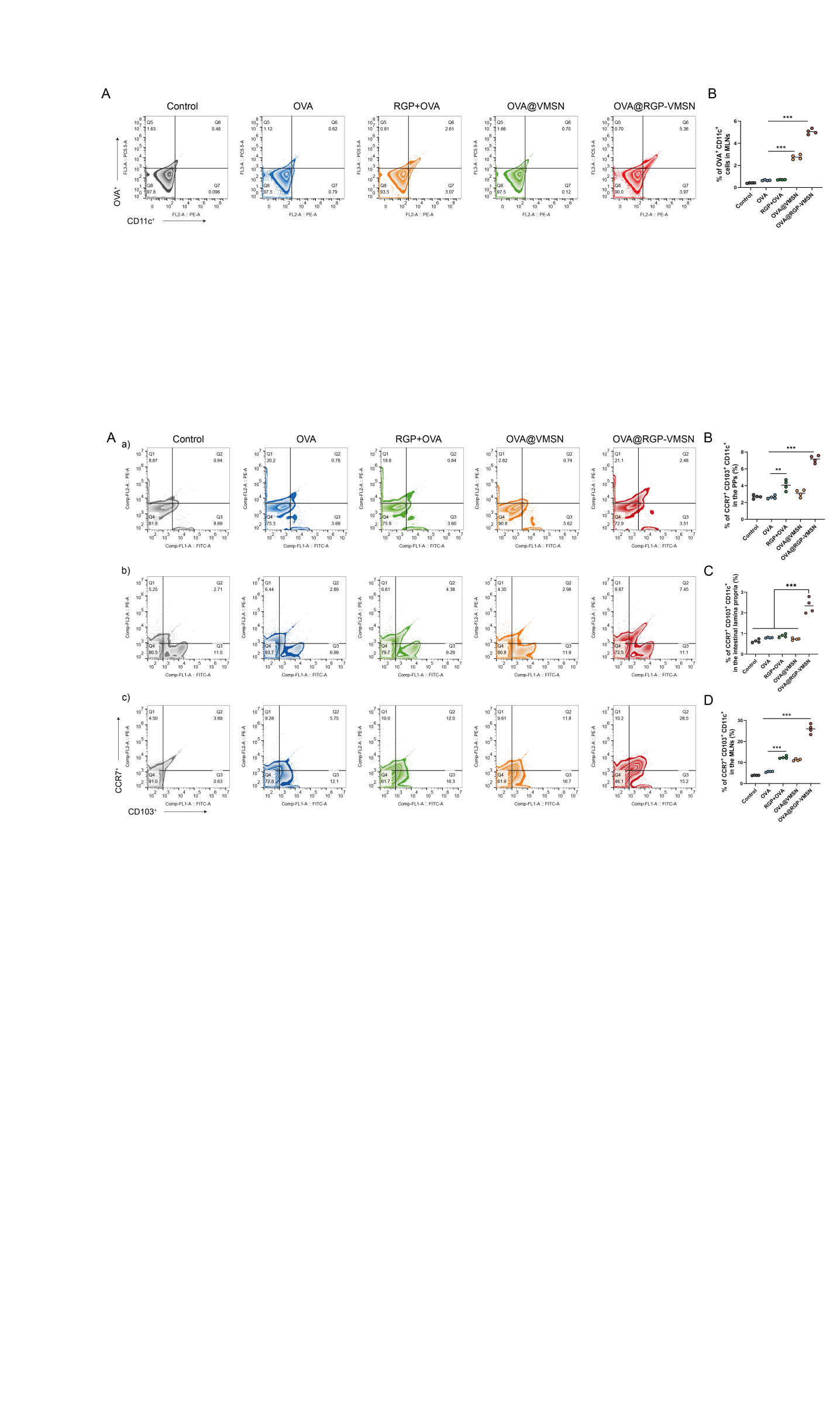


**Fig. S17.** At 12 h after oral administration, (A)FCM plots showing the proportion of OVA^+^ cells among CD11c^+^ DCs in MLNs at 12 h after oral administration. (B) Quantitative analysis of the proportion of OVA^+^ cells among CD11c^+^ DCs in MLNs. Each symbol represents an individual sample (n = 4 mice per group). Data are presented as mean ± SD. ****P* < 0.001 by one-way ANOVA followed by Tukey’s multiple-comparison test.


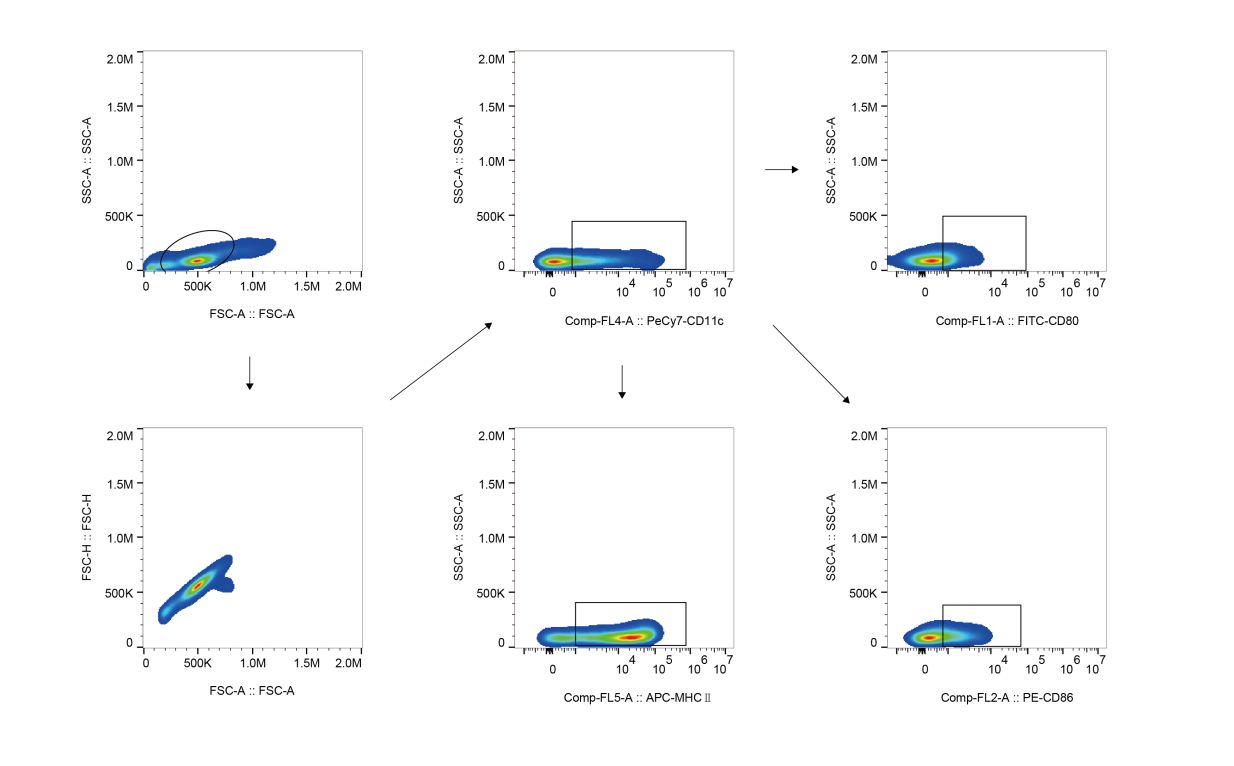


**Fig. S18.** Flow cytometry gating strategy for analyzing CD80, CD86, and MHC-II expression in CD11c^+^ cells from lymphnodes.


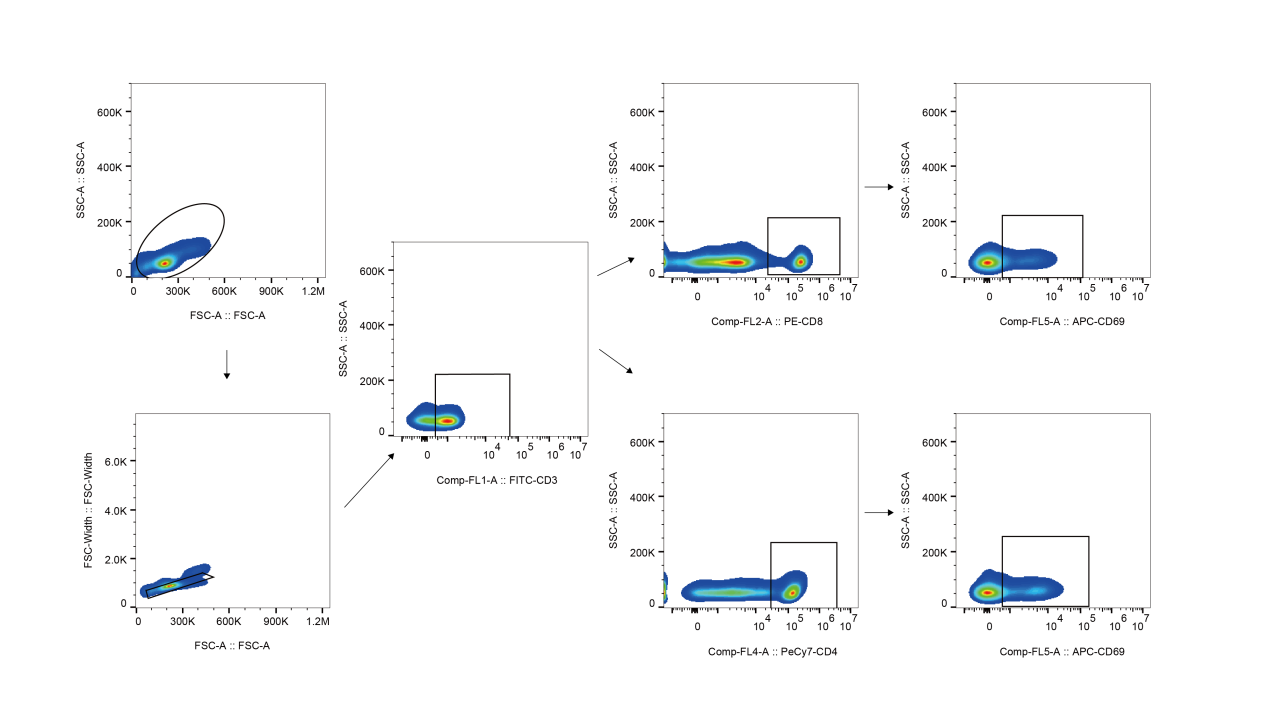


**Fig. S19.** FCM gating graphs relating to the T-cell activation in LNs.


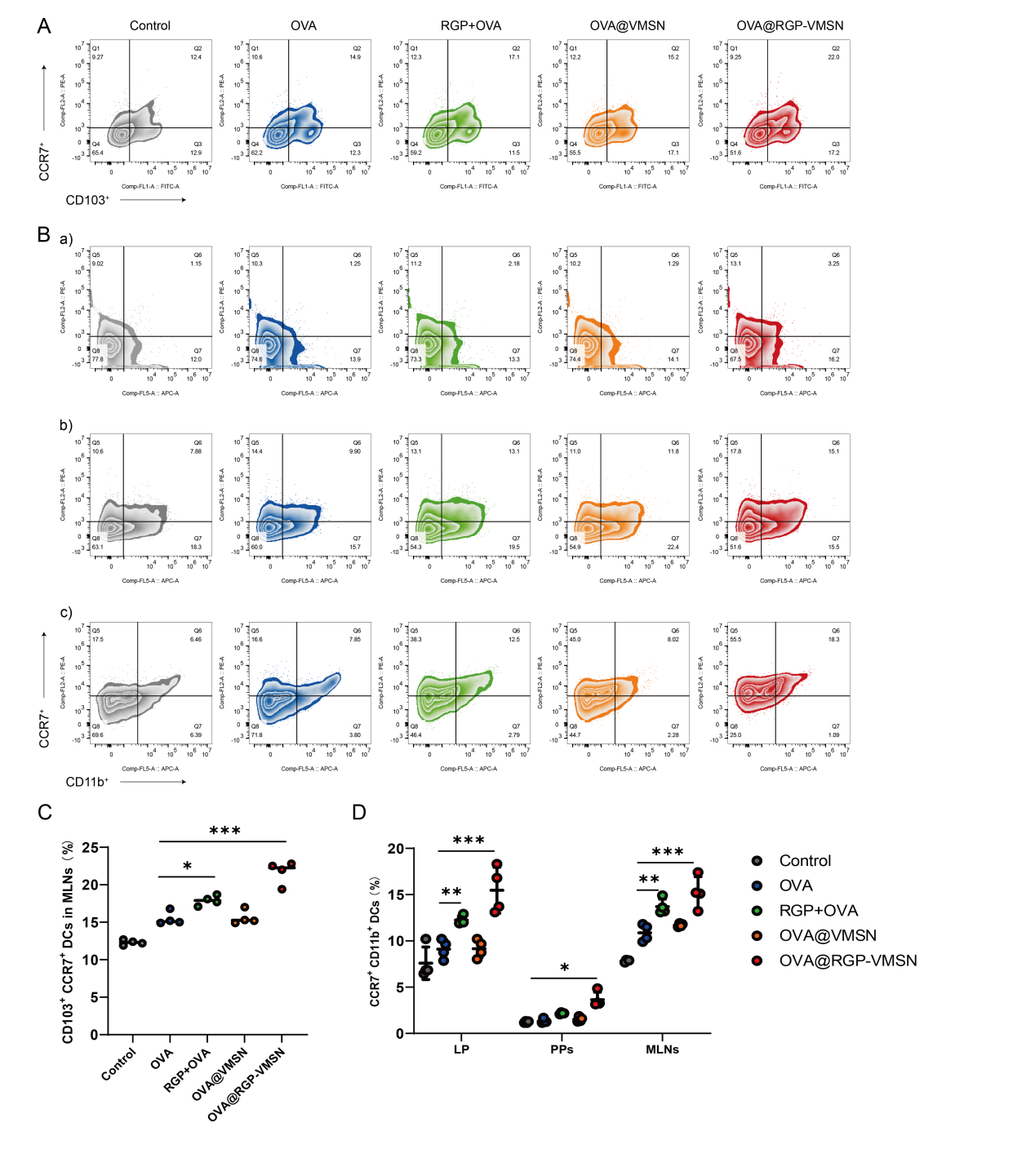


**Fig. S20.** At 24 h after oral administration, (A)Flow cytometry plots of CCR7^+^CD103^+^CD11c^+^ DCs in MLNs.(B) Flow cytometry plots of CCR7^+^CD11b^+^CD11c^+^ DCs in (a) PPs, (b) LP, and (c) MLNs.(C) Quantitative analysis of CCR7^+^CD103^+^CD11c^+^ DCs shown in MLNs.(D) Quantitative analysis of CCR7^+^CD11b^+^CD11c^+^ DCs shown in PPs, LP, and MLNs.

Each symbol represents an individual sample (n = 4 mice per group ). Data are expressed as means ± SD. **P* < 0.05, ***P* < 0.01, ****P* < 0.001 by one-way ANOVA followed by Tukey’s multiple-comparison test.


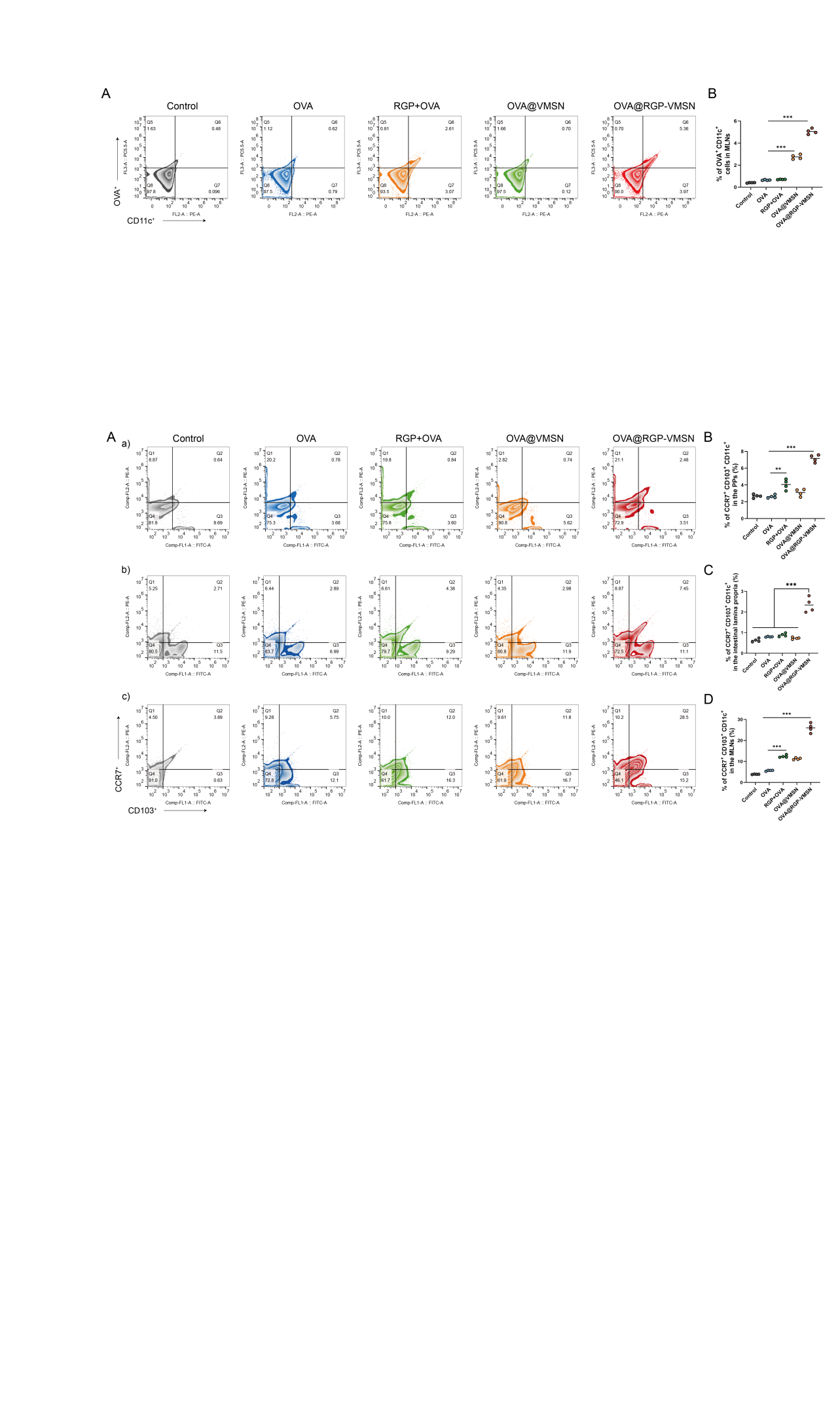


**Fig. S21.** At 48 h after oral administration, (A) Flow cytometry plots of CCR7^+^CD103^+^CD11c^+^ DCs in (a) PPs, (b) LP, and (c) MLNs; Quantitative analysis of CCR7^+^CD103^+^CD11c^+^ DCs shown in (B) PPs, (C) LP, and (D) MLNs. Each symbol represents an individual sample (n = 4 mice per group). Data are expressed as means ± SD. ***P* < 0.01, ****P* < 0.001 by one-way ANOVA followed by Tukey’s multiple-comparison test.


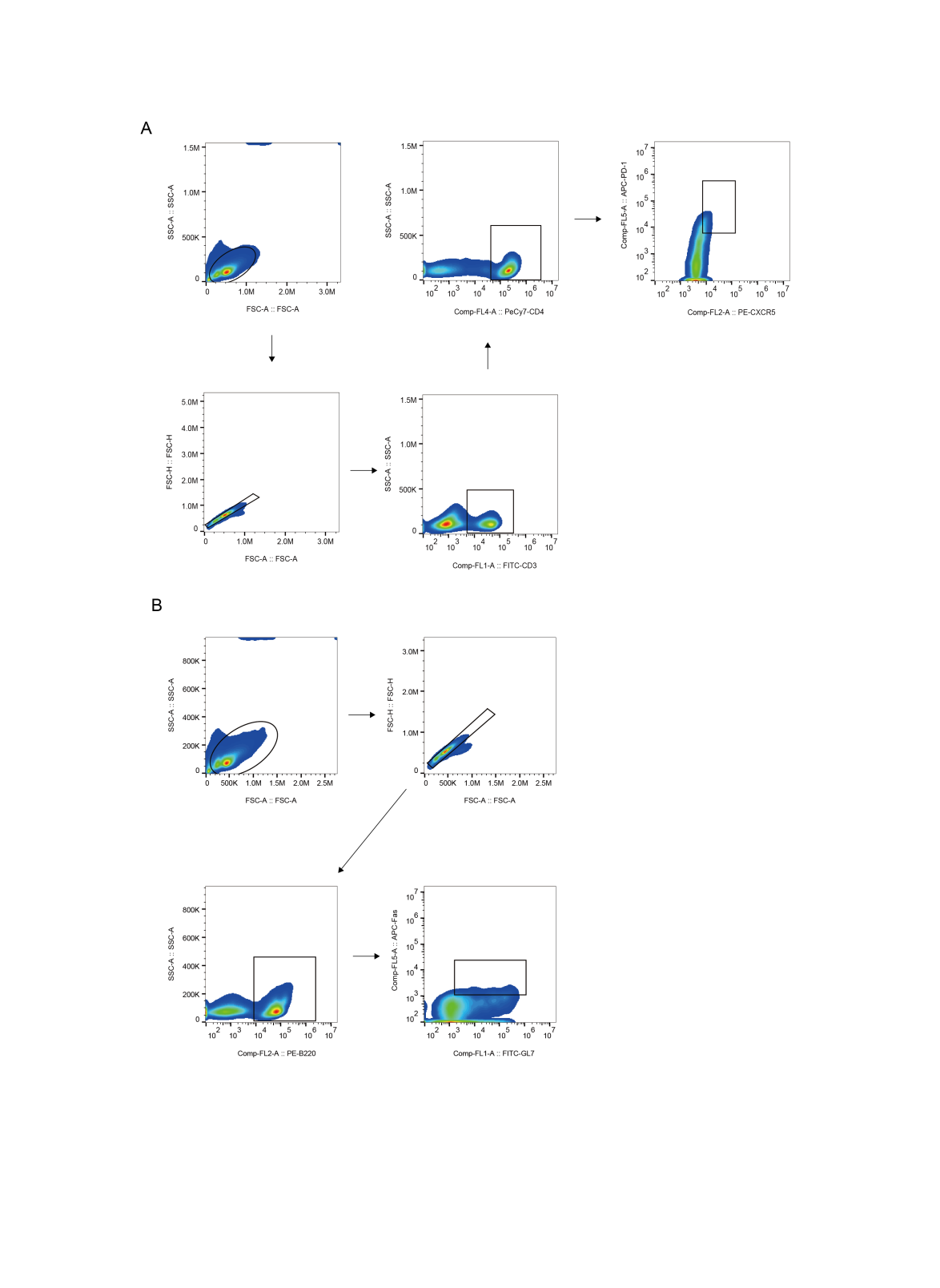


**Figure S22.** FCM analysis of gating strategies to determine the analysis of Tfh cells (A), GC B cells (B) in the LNs.

**
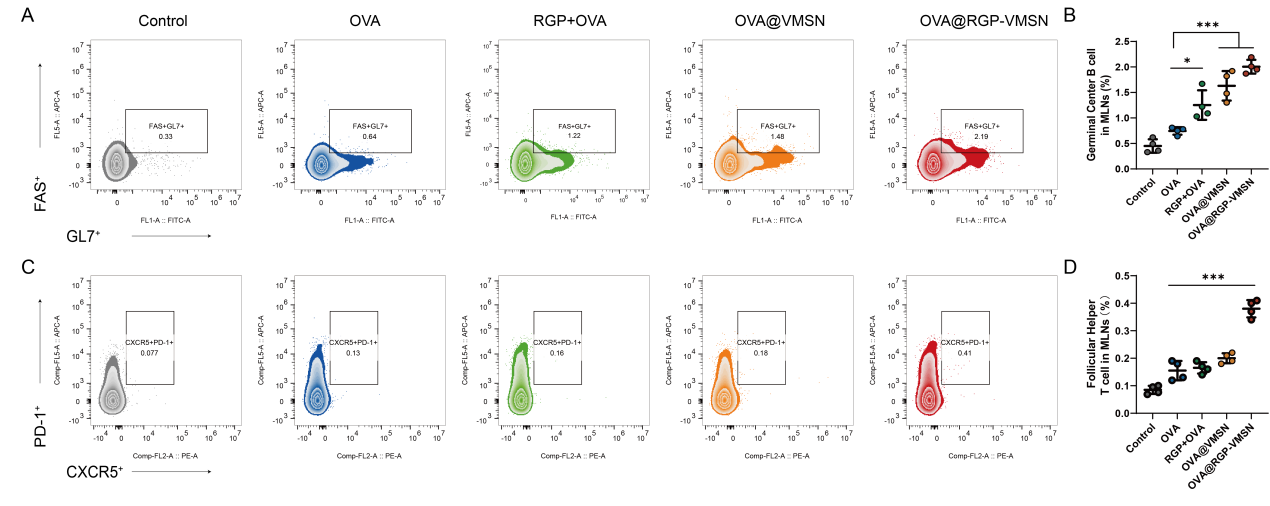
**

**Fig. S23.** (A,C) Tfh and GC B cells in MLNs are shown in representative FCM plots.(B,D) Quantification of Tfh cells and GC B cells in MLNs.Each symbol represents an individual sample ( n = 4 mice per group). Data are presented as mean ± SD.*** *p* < 0.001, * *p* < 0.05 by Tukey’s multiple-comparison test.


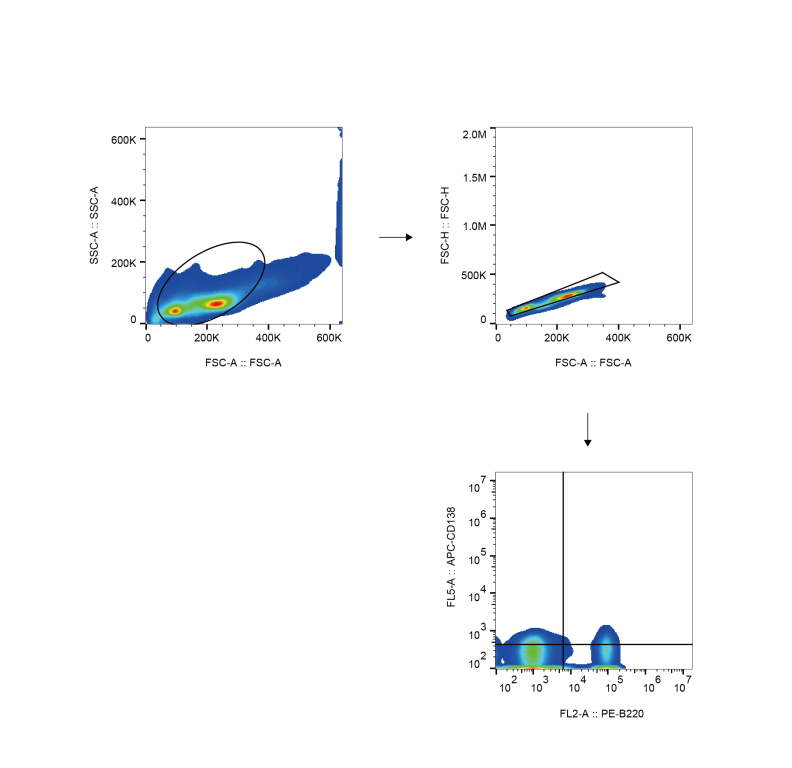


**Fig. S24.** FCM analysis of gating strategies to determine the analysis of plasma B cells in MLNs.
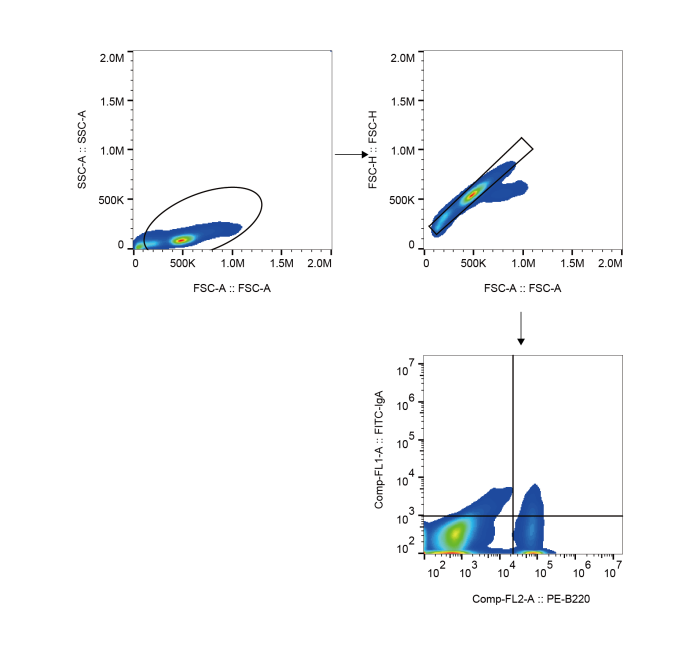


**Fig. S25.** FCM analysis of gating strategies to determine the analysis of IgA^+^ B cells in MLNs.


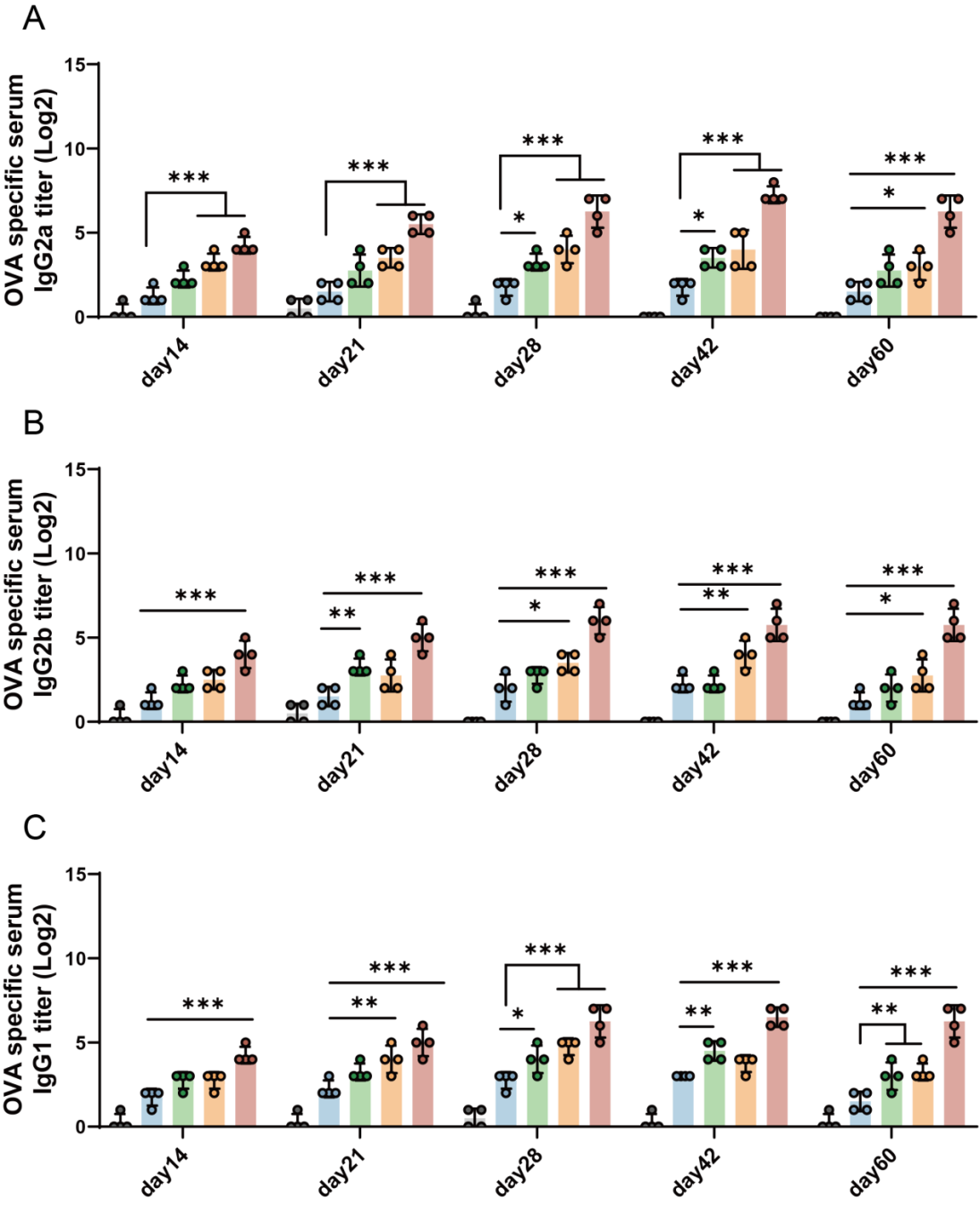


**Fig. S26.** (A-C) Time-course of OVA-specific IgG2a, IgG2, IgG1 titers in serum after oral immunization. Each symbol represents an individual sample ( n = 4 mice per group ). Data are presented as mean ± SD. *** *P* < 0.001, ** *P* < 0.01, * *P* < 0.05 by Tukey’s multiple-comparison test.


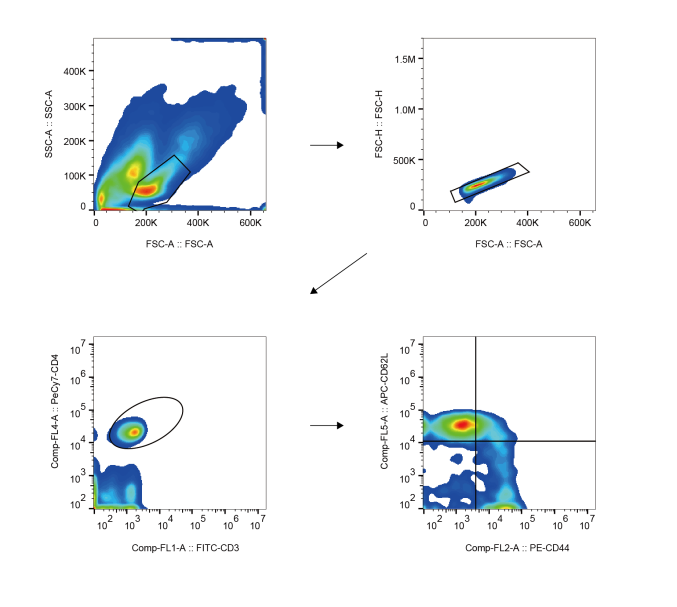


**Fig. S27.** FCM gating graphs relating to the splenic memory CD4^+^ T cell


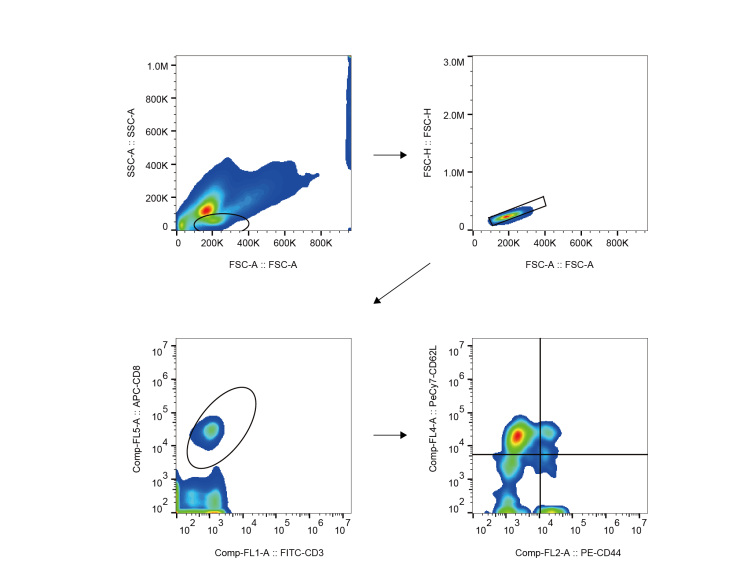


**Fig. S28.** FCM gating graphs relating to the splenic memory CD8^+^ T cell


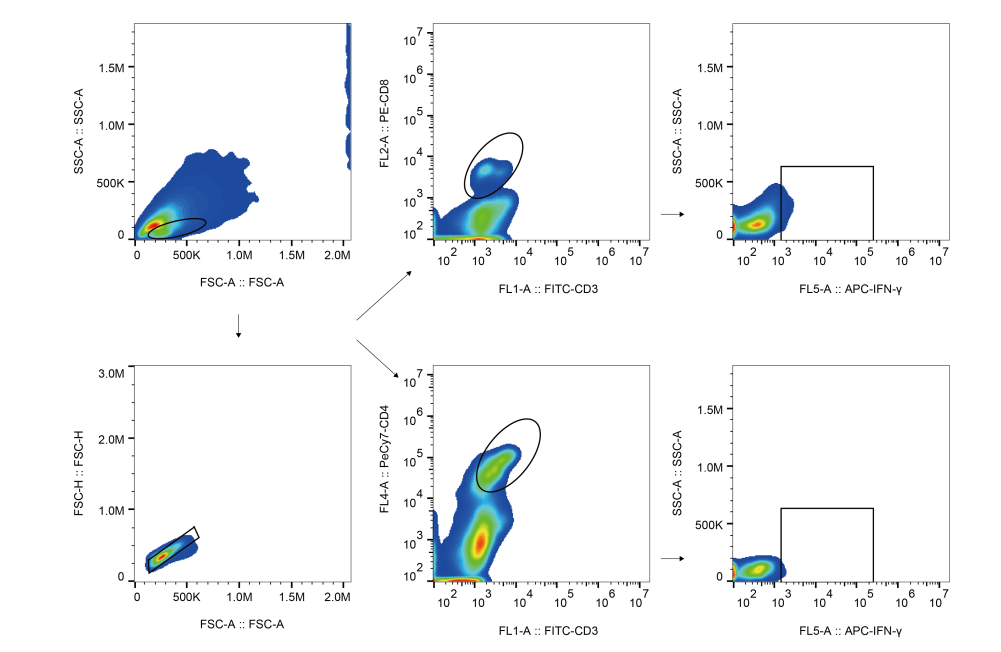


**Fig. S29.** Gating strategies of IFN-γ-positive cells in CD4^+^ and CD8^+^ T cells.

***
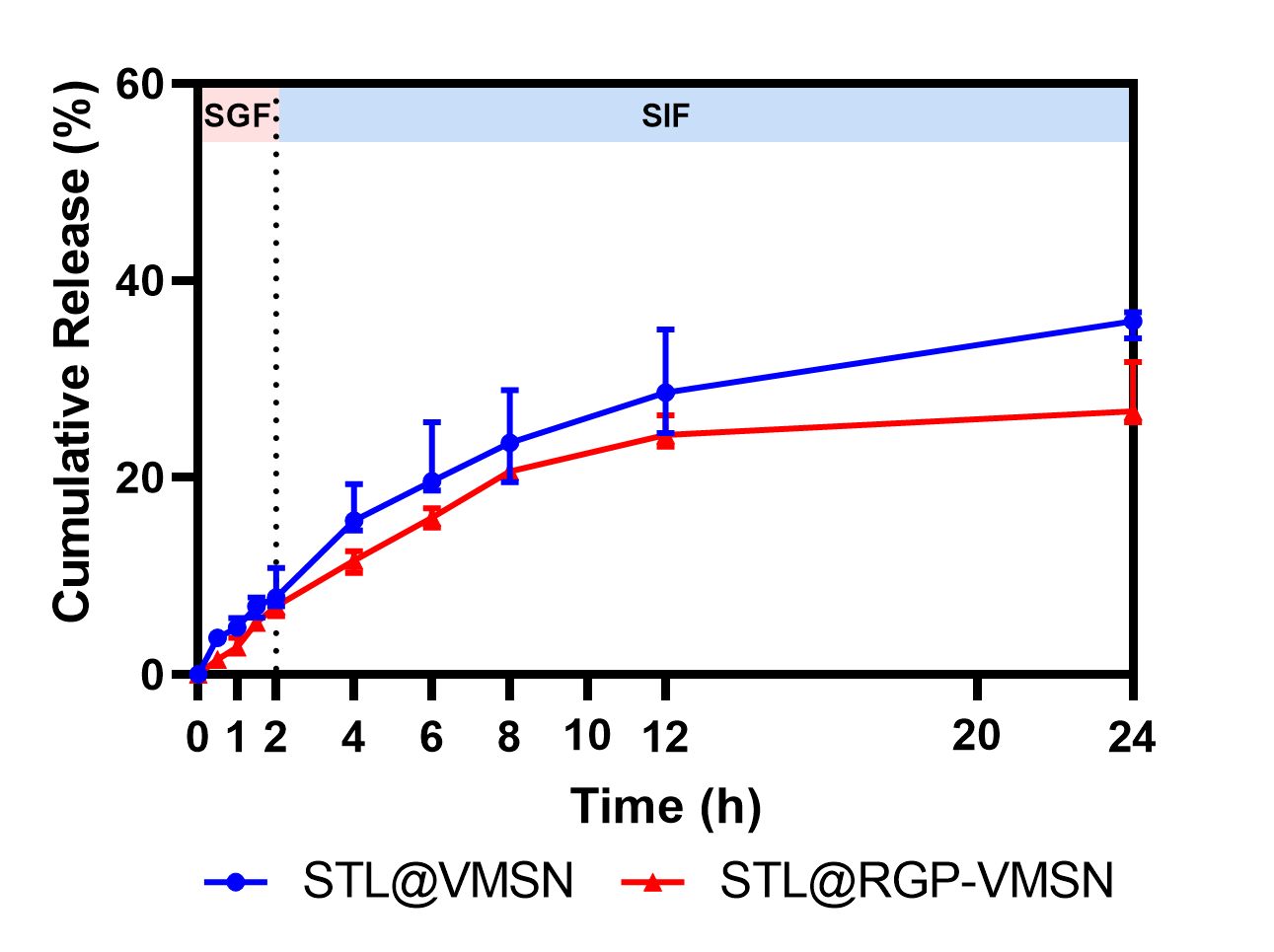
***

**Fig.S30.** The cumulative release profiles of STL from VMSN and RGP-VMSN in SGF (without pepsin) and SIF (without trypsin) at 37 ℃. Data are expressed as means ± SD (n = 3 independent experiments).


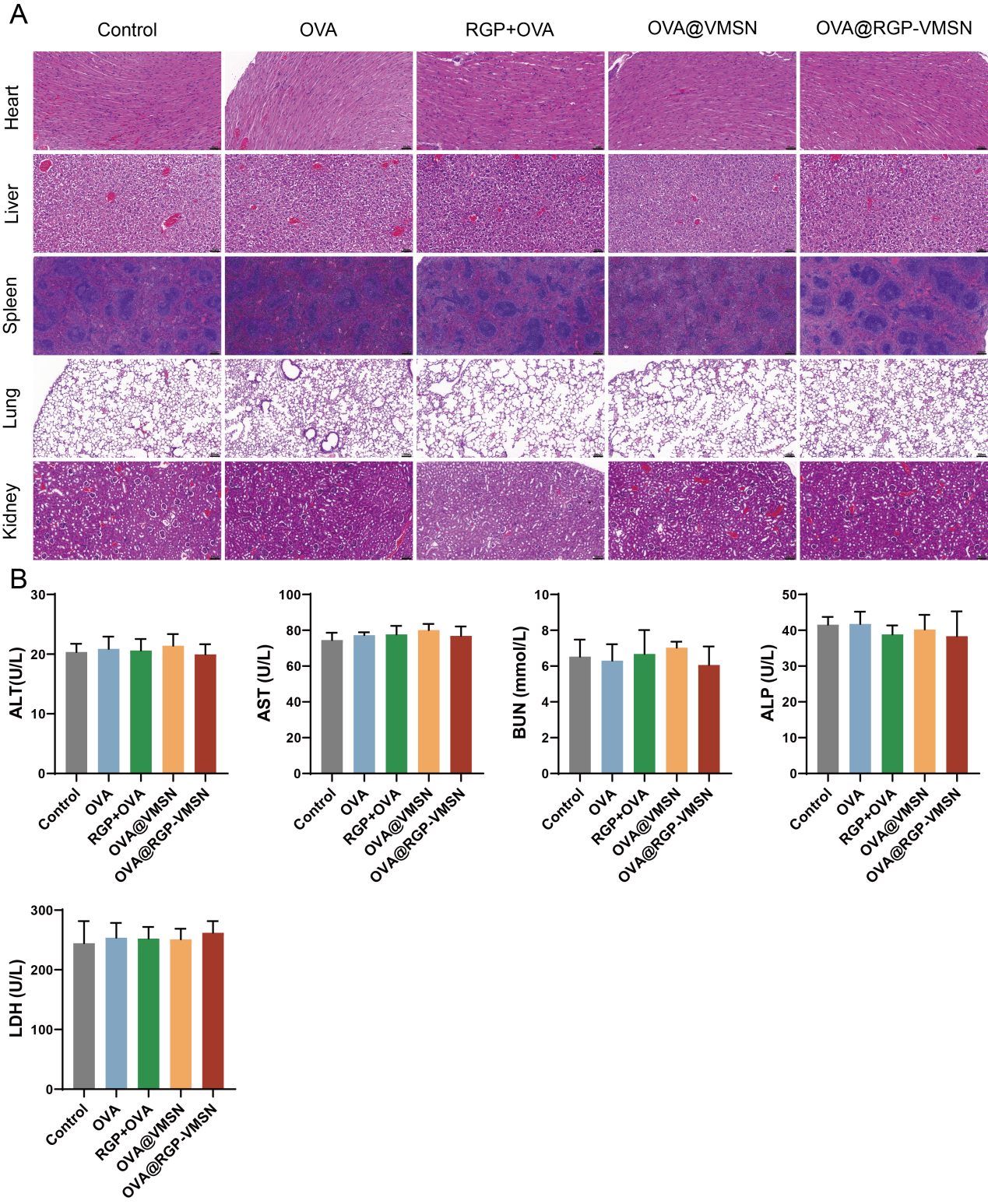


**Fig. S31.** (A) H&E-stained sections of major organs (heart, liver, spleen, lung, and kidney) collected from mice on day 60 after initial immunization. Scale bars, 50μm All images are representative of n = 4 mice per group.(B) Serum biochemical parameters including alanine aminotransferase (ALT), aspartate aminotransferase (AST), blood urea nitrogen (BUN), alkaline phosphatase (ALP), and lactate dehydrogenase (LDH), measured on day 60 after initial immunization. Data are presented as mean ± SD from 4 mice per group.


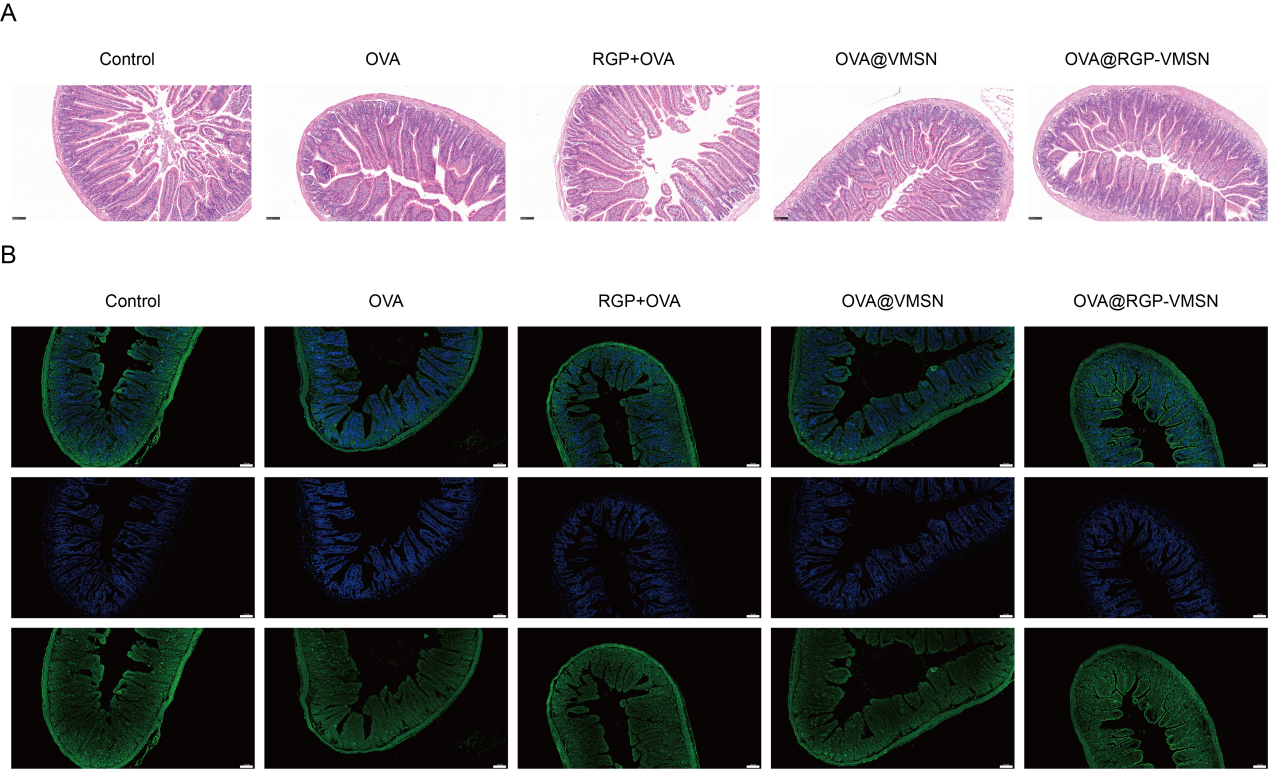


**Fig.S32.** (A) Representative images of H&E-stained intestinal tissues collected from mice on day 60 after initial immunization. (B) Immunofluorescence staining of ZO‑1 (green) and nuclei (blue, DAPI) in intestinal tissues collected from mice on day 60 after initial immunization. Scale bar: 100 μm. All images are representative of n = 4 mice per group.
